# Thin flexible lab-on-a-film for impedimetric sensing in biomedical applications

**DOI:** 10.1038/s41598-022-04917-5

**Published:** 2022-01-20

**Authors:** Amina Farooq, Fezan Hayat, Sobia Zafar, Nauman Zafar Butt

**Affiliations:** 1 Department of Electrical Engineering, Lahore University of Management Sciences, Lahore, Pakistan; 2 Thunderbird, Arizona State University, Tempe, USA; 3 SEECS, National University of Science & Technology, (NUST), Islamabad, Pakistan

**Keywords:** Biophysics, Biotechnology, Cancer, Developmental biology, Diseases, Health care, Medical research, Engineering, Materials science, Physics

## Abstract

Microfluidic cytometers based on coulter principle have recently shown a great potential for point of care biosensors for medical diagnostics. Here, we explore the design of an impedimetric microfluidic cytometer on flexible substrate. Two coplanar microfluidic geometries are compared to highlight the sensitivity of the device to the microelectrode positions relative to the detection volume. We show that the microelectrodes surface area and the geometry of the sensing volume for the cells strongly influence the output response of the sensor. Reducing the sensing volume decreases the pulse width but increases the overall pulse amplitude with an enhanced signal-to-noise ratio (~ max. SNR = 38.78 dB). For the proposed design, the SNR was adequate to enable good detection and differentiation of 10 µm diameter polystyrene beads and leukemia cells (~ 6–21 µm). Also, a systematic approach for irreversible & strong bond strength between the thin flexible surfaces that make up the biochip is explored in this work. We observed the changes in surface wettability due to various methods of surface treatment can be a valuable metric for determining bond strength. We observed permanent bonding between microelectrode defined polypropylene surface and microchannel carved PDMS due to polar/silanol groups formed by plasma treatment and consequent covalent crosslinking by amine groups. These experimental insights provide valuable design guidelines for enhancing the sensitivity of coulter based flexible lab-on-a-chip devices which have a wide range of applications in point of care diagnostics.

## Introduction

Flexible lab-on-a-chip (LOC) devices have rapidly intrigued the scientific community interest due to their possible utility in health monitoring and medical diagnostics^1–5^. The biosensors are based on the specific interaction of a biological recognition factor with the target biomolecules and a compatible transducer that tracks the degree of this interaction and provides qualitative and quantitative data. When used as support platforms for biosensors, flexible materials have many appealing characteristics: lightweight, ultra-conformable (bendable, foldable, stretchable), compact, disposable, and affordable. Moreover, modular devices allow for a greater degree of functional integration and, as a result, can manage additional features such as controlled data acquisition instrumentation, wireless transmission modules, and in-built power units, etc.^6–8^. The demand for flexible sensors is projected to hit $8 billion by 2025. Flexible biosensors will be among the fastest-growing flexible sensing devices in the next decade, with more than 40% CAGR^5,9^.

Flexible electrodes in amperometric, field-effect transistor (FET)-based potentiometric, voltametric, and impedimetric electrochemical detection modes are ideal for versatile biosensing systems^10^. These electrodes can be easily miniaturized and can be produced using various fabrication techniques (such as screen printing or sputtering). These transducers can tolerate extreme mechanical deformations (bending, folding, stretching) with minimal loss of sensing efficiency. Furthermore, in biosensing, electrical detection allows for high selectivity and sensitivity. Lighter, flexible, and smaller electronics have become significant electronic devices due to the growing demand for multifunctional digital sensors^10^. It is one of the most extensively researched areas of many of these prospective directions, and flexible electronics has piqued the interest of both the industrial and academic sectors. The use of flexible electrodes in next-generation flexible electronics, such as touch screens, adjustable lighting, flexible solar cells, and wearable sensors, has sparked an enormous surge^11^. Unlike conventional rigid and heavy devices, flexible electronic devices can change their shape to meet the needs, making them more compact, functional, and perhaps even convenient. The rise of flexible devices has escalated the research into flexible electronic components in portable electronic devices^12^.

The development of portable microfabrication technologies has resulted in the development and fabrication of microelectrodes with various structures and functions for biomedical research, diagnosis, and treatment through electrical stimulation and electrophysiological signal recording. Kassal et al. proposed an amperometric bandage-based system with wireless connectivity to a smartphone for uric acid biosensing to measure wound status^13^. Beyond rigid microelectrodes made of silicon or glass, flexible microelectrodes have many advantages, including lighter weight, smaller length, better skin conformance, and lower fabrication costs^14^. Low material consumption and sometimes low-cost materials distinguish Lab-on-a-Film devices, making them appealing for cost-effective, high-volume manufacturing of self-contained portable devices^15^. Glass has been the most popular substrate material so far but can have practical issues during structuring and bonding, and is not suitable for wearable applications^16,17^. Polymers, on the other hand, have low material cost^18,19^ and can easily be integrated with microstructures using a variety of proven techniques, ranging from lithography^20,21^ with nm-scale dimensions to a simple bonding method^22,23^, all of which can be done in a typical laboratory setting. Besides being light weight, the polymer structures are conformable to the curved edges of the human body and versatile to body movements. These practical benefits signifies the need to develop high performance and robust fabrication technologies for flexible substrates^24^. A list of representative applications of flexible LOC platforms on plastic substrate is summarized by Economou et al.^1^.

Conventional biomedical devices include microfluidic channels and detection electrodes both of which have micrometer scale dimensions. The fluidic channels are usually made of soft materials, namely, PDMS, and the suspension mediums are generally liquid^6^. A completely flexible device however requires the substrate with printed electrodes to be flexible. Flexible electrode substrates are now commonly used in a broad range of electronic devices. To adjust with the shape or form of the human body, flexible substrates have become essential components for wearable devices^25–29^. The wide range of physical and mechanical properties exhibited by different plastics (e.g., weight, moldability, durability, and gas- and water-permeability) have occupied a central position as frontline products with special plastics solving problems specific to those local industries. Among various flexible polymers, polypropylene (PP) has rapidly attracted a large interest due to its low density and excellent chemical resistance among commodity plastics. Since PP belongs to the semi-crystalline group, crystallinity is usually between 40 and 60%^24,30^. PP is a low-cost thermoplastic polymer with excellent flame resistance, clarity, heat distortion temperature, dimensional stability, and recyclability, making it suitable for various applications^1,6^. Thermal stability is an important requirement for the flexible substrates that are used in point of care microfluidic devices. PP has a higher melting point than polyethylene, which makes it suitable for the applications in food and medicine industry that requires sterilization at high temperatures. These properties are the trademarks for PP film application in various fields such as food and medical industry^31–33^. For the cell counting device, the flexibility can enable the use for wearable complete blood counting. Moreover, by integrating a receptor layer with cell counting, a large variety of wearable immunoassays can be implemented for continuous measurements in a variety of applications including detection of pathogens, disease biomarkers, and tumor cells.

Here we explore some of the important design considerations for flexible microfluidic cell counter made on PP substrate. The position and geometry of the microelectrodes is varied to optimize the signal to noise ratio. The paper is organized in sub-sections. Section II describes the materials and methods inclusive of the design of coplanar microelectrodes and the microchannel for the modified layout (Design M) in comparison with the standard (Design S), biochip fabrication steps, alignment, bonding, and the electrical set up for data extraction. The results for the physical characterization and electrical cell counting are compiled in Section III. A brief summary and the conclusions of the paper are provided in section IV.

## Materials and methods

We have developed a simple method of producing a flexible electrode thermally resistant plastic film. The first step is the photolithography of a microscale pattern of the electrodes on polypropylene film to obtain a patterned-electrode film using a liftoff process. This is followed by the magnetron sputter deposition to fabricate multilayered metals on the film. The bonding between microfluidic channels in PDMS with microelectrodes metal structures on thin polypropylene film is optimized through the surface modification method.

### Computational design

The geometrical electrode configuration was first optimized in COMSOL Multiphysics software in our previous paper^34^. Notably, electrode geometry, i.e., width, position, and size of the gap between the electrodes, were optimized. The standard approach for the coplanar electrodes is to place these at each side of the confined detection volume at the bottom of the microchannel that is in contact with the substrate. A key design modification is the repositioning of the microelectrodes under the sensing zone for better focusing of the electric field into the constriction zone in the detection volume as clearly illustrated in Design S for the standard and Design M for the modified layout in the Supporting Information Fig. S1a,b. The sensitivity improvement of the modified approach has been predicted in our previous paper using COMSOL^34^. Here, we do the comparative experimental study between the biochips made of Design S with that made of Design M.

### Photomask design layout

**Figure 1:**
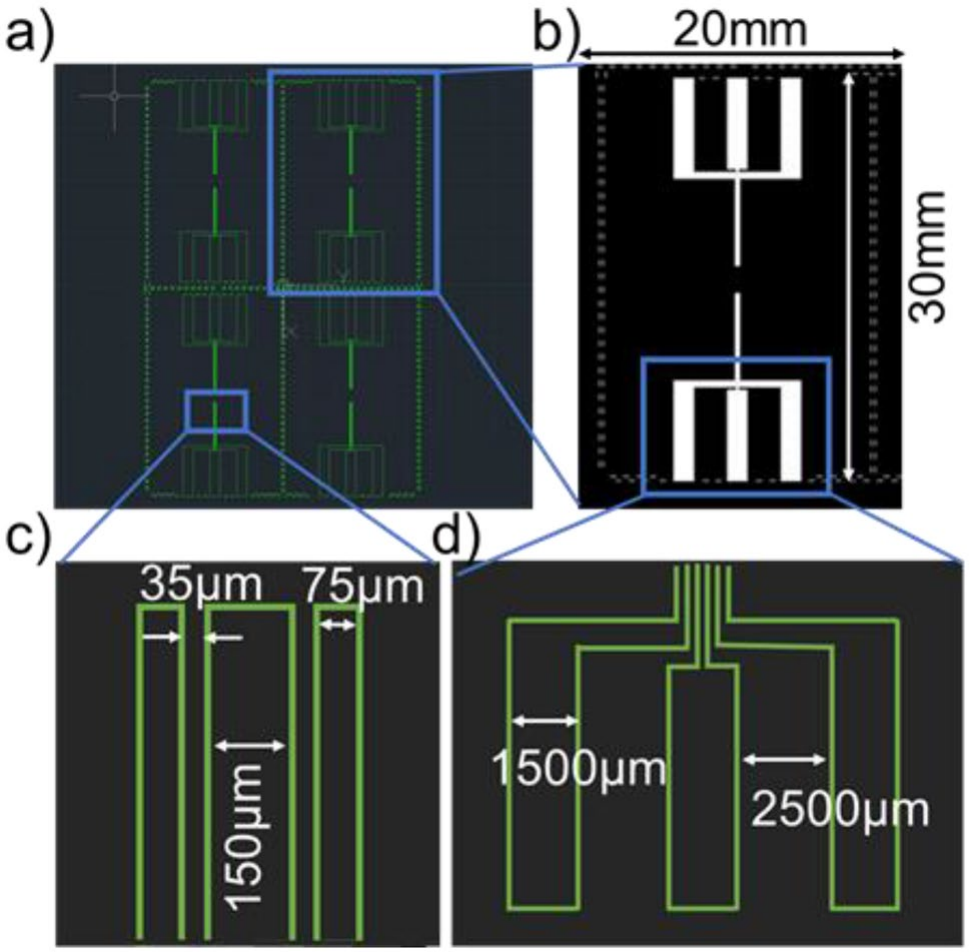
Design M: (**a**) 4 × 4 inches microelectrodes photomask layout on AutoCAD, (**b**) Isolated microelectrode for single biochip, (**c**) Coplanar electrodes dimensions and the gap between them, (**d**) Extended electrodes area dimensions for soldering on PCB or external setup.

**Figure 2:**
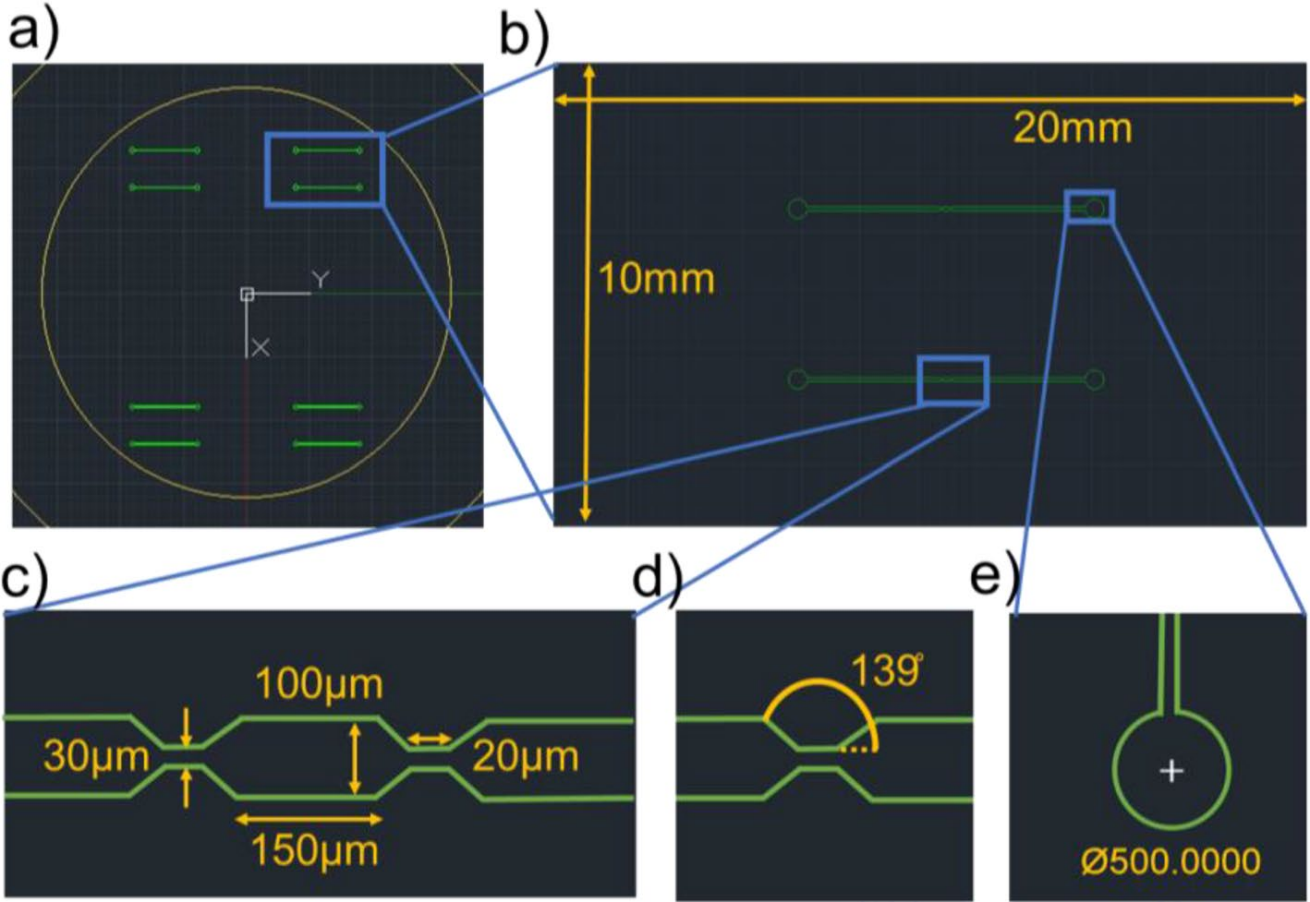
(**a**) The AutoCAD layout of microfluidic channel photomask for Design M biochip fabrication, (**b**) Single final chip microchannels with 20 × 10 mm length × width, (**c**) The detailed dimensional explanation of constricted microchannel mask layout, (**d**) The inclination angle between a microfluidic channel and the constricted channel is 139°, (**e**) The inlet and outlet hole diameter.

Photomask for patterning microfluidic channels and the microelectrodes is drawn in AutoCAD software^35^. The total surface area of the device is 30 mm × 20 mm for Design M. The device size was chosen to match its performance as a flexible chip, which increases its commercial importance when developed. Detailed layout of the transparency mask for Design M is shown in Fig. 1. The length of the central electrode is set to 150 µm with an adjacent electrodes' size at 75 µm and a 35 µm separation between them. The extended portion of all electrodes for soldering with an external setup consists of 1500 µm length and 2500 µm gap as displayed in Fig. 1d. Figure 2 shows the constricted microchannel architecture layout developed on a transparency mask for Design M. The microfluidic channel has an inlet and outlet for the fluid flow. The dimensions of microfluidic channels are optimized according to the microelectrode's layout. The integrated device contains two microfluidic channels aligned and bonded with two microelectrodes for impedimetric detection. The total photomask area is 4 × 4 inches, and the transferred pattern area is inscribed within 3-inches for substrate surface. The total length and width of the single microfluidic chip containing two parallel channels are 20 mm and 10 mm, respectively.

The overall channel width is maintained at 100 µm, facilitating the capillary effect for fast transportation of fluid through microfluidic channels. Both sensing areas are 20 µm and 30 µm length and width, respectively, positioned precisely at the inlet and outlet hole center. The central portion is 150 µm long and at similar width of 100 µm as the microfluidic channel. The 35 µm long tilt is created, which unites constricted areas with the microfluidic channel on both sides. The size of both inlet and outlet is the same dimension at 500 µm as in Fig. 2e.

The system dimensions were chosen to increase the chip's sensitivity in the sub-micrometer range using standard photolithography while still detecting larger particles (up to 30 μm). Theoretically, extending the electrodes in the channel direction would result in a higher current between the electrodes. However, it is not beneficial to significantly increase the electrodes' width since this results in inconveniently long particle transition times resulting in flow of multiple cells at a time within the detection volume^34^. The width of the middle electrodes is from 100 to 150 μm for both the standard (Design S) and modified (Design M) design, this was discovered to be a good balance of signal strength and transition length. In new layouts, the distance between electrodes is set to 35 µm, which offers high sensitivity while maintaining a large enough detection volume for larger particles. The chosen distance between the electrodes and electrode lengths ensures that the device can analyze single particle to a concentration of approximately 10^7^ particles/mL^34^. The microfluidic channels in the Design S are 200 μm wide and 50 μm high. The coplanar electrodes exposed to the track in the standard chip are 100 μm wide (w) with a gap of 100 μm (see Supporting Information Figs. S2 and S3). While for the Design M, the periphery electrodes are 75 μm wide (w) with an inter-electrode distance of 35 μm^36^.

### Microelectrode and microchannel fabrication

The designs are executed for lab-on-chip fabrication combining both microfluidics and microelectrodes. The electrodes were fabricated through a standard photolithography and liftoff process in a cleanroom. Substrate cleaning is the preliminary step in the microfabrication processes. The next step is a UVO treatment to a thin polypropylene film and a supporting glass plate for film attachment on the hard surface. This step helps in eliminating discomfort in polymer thin film handling during the photolithography process. The positive photoresist (AZ1512 up to 2 µm) is spun on a polypropylene film at 2800 rpm for 45 s and cured on a hotplate at 100 ºC for 70 s. The photoresist is patterned with a UV exposure energy dose of 34 mJ/cm^2^ and is developed (MIF-300, AZ electronic materials) for 20 s. A 100 nm of platinum is then sputtered on it with a 30 nm titanium adhesion layer via magnetron sputter. After the Ti/Pt layer is deposited, the attached polypropylene film with glass supporting substrate is inserted in a solvent to remove photoresist and aid in detachment. The precise and reliable metal patterns are fabricated on the polypropylene (PP) substrate (see in “Results and discussion” section). These metal patterns define the design of the electrode and the device outline. Finally, individual pair of electrodes for a single biochip is separated using a razor (displayed in “Results and discussion” section).

The microfluidic channel layer is created using a silicon mold for PDMS via photolithography and soft lithography technique. The mold is fabricated through a photolithography process, starting with a silicon wafer (thickness 380 µm) cleaning step. The wafer is then coated with a negative photoresist (SU-8 100 µm) at 700 rpm for 45 s and pre-bake on a hotplate at 65 °C for 15 s and 95 °C for 2 h. with the ramping rate of 2 °C per minute. The SU-8 is then exposed to UV energy with 204 mJ/cm^2^ dose and post-exposure bake at similar temperatures for 15 and 45 min. It is developed in PGMEA for 04 min, followed by washing in IPA and DI water. It is then followed by drying the patterned SU-8 on a silicon wafer with N_2_-gun and hard bake at 130 °C for 2 h.

**Figure 3:**
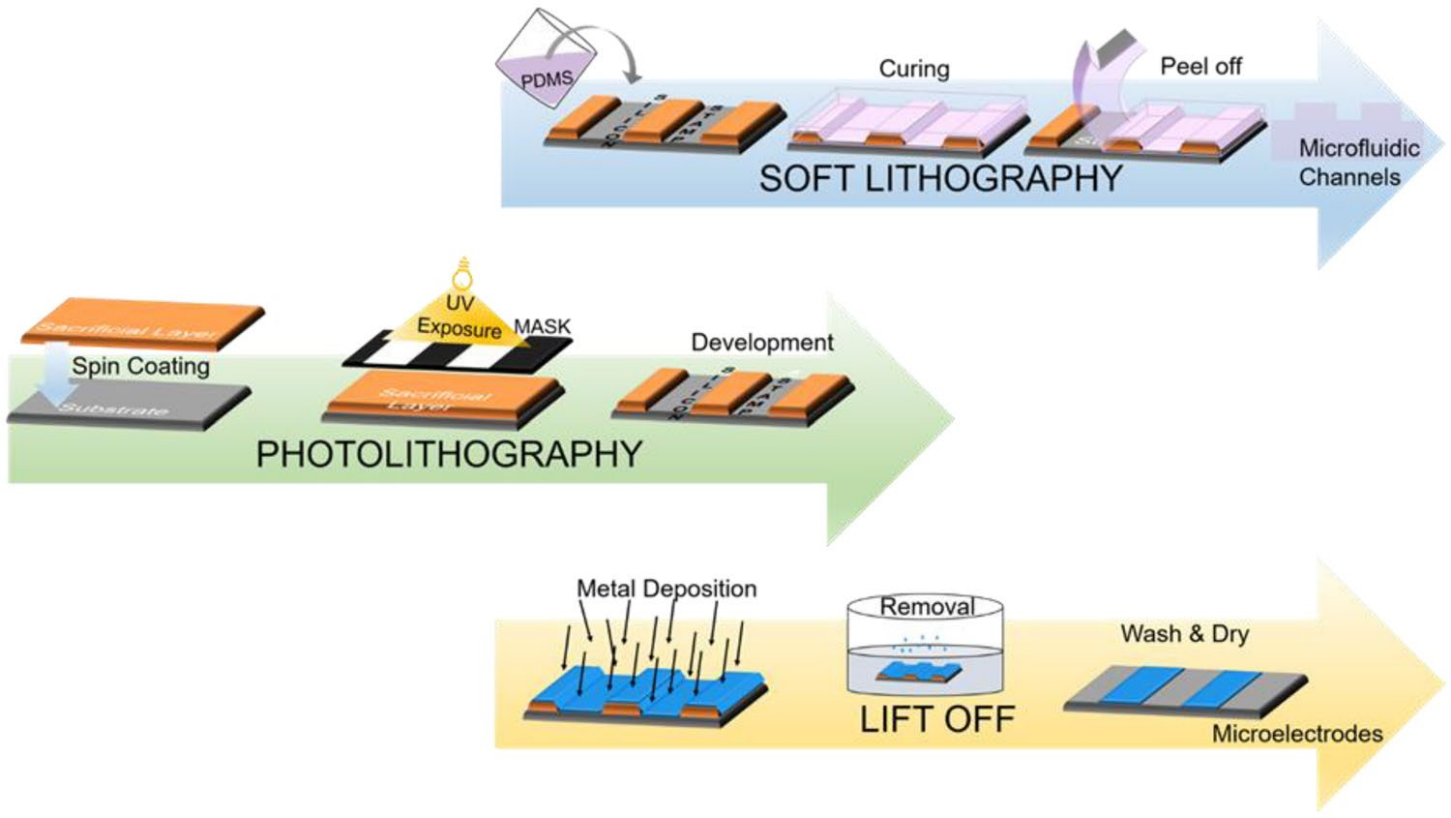
Illustration of three fabrication techniques for microelectrodes and microchannel development for the lab-on-a-film device. The photolithography process is used for the SU-8 created stamp on silicon substrate for microchannels fabrication. Soft lithography is adopted for the pattern transfer from silicon stamp into PDMS layer. And liftoff process is done for the microelectrode's formation on a polypropylene film through a metal deposition.

The next step is the preparation of PDMS microchannels via the soft lithography process. It is done by mixing the curing agent and PDMS monomers in a 1:10 ratio in a disposable bowl. We use a mixture that consists of 0.9 g of curing agent and 9 g of monomer. PDMS is subsequently poured on the silicon wafer containing SU-8 defined microchannels, cured at 80 °C for 1 h. PDMS slabs are peeled off the SU-8 master stamp and the inlet and outlet holes are punched. The stepwise description of all three processes of photolithography, soft lithography, and liftoff is explained schematically in Fig. 3. The PDMS channels are aligned afterward with the electrodes using a mask aligner.

### Surface modification, alignment and bonding

Surfaces can be modified when desired surface properties are needed. Changing wettability can boost or stifle capillary liquid flow^37,38^. Surface modifications also facilitate bonding^39–41^, allowing microfluidic chips to be sealed more effectively. Plasma treatment, UV irradiation^42^, and laser sources are all commonly used methods for surface modification^43^. These approaches often employ inserting functional groups into the surface layer, allowing for functionalization in several applications.

We modify PP and PDMS surfaces before permanent bonding & sealing for biomedical applications. Both the PP and PDMS are treated under various conditions, including (i) oxygen plasma treatment (100 W, 2 min), (ii) UVO treatment (15 min), (iii) air plasma treatment (RF, 10 min), (iv) oxygen plasma treatment (100 W, 2 min) and then treatment with 1% v/v aqueous APTES solution for 20 min at 60 °C; and (v) immersing in an aqueous solution of 1% v/v APTES for 20 min without prior oxygen plasma treatment. After immersion in an APTES solution, the membrane surfaces are terminated with hydrophilic amino groups such as a silanol group between two molecules, a hydroxyl group on the top, or an amine group influential in the hydrogen bond^41,44–57^.

All the substrates treated with amino silane treatment are then washed with DI water and blown dry with an N_2_ gun. Different combinations of the activated PP and PDMS substrates are kept in contact at room temperature for an hour, and bonding is evaluated by a manual peeling test and high-rate liquid pressure flow. The results are summarized in the Supplementary Information, SI Tables S1 & S2. At each surface treatment phase, the wettability of the substrate surface, or the contact angle of water droplets, is calculated and is shown in the SI Table S1.

The microelectrode patterned PP film is aligned with microchannel defined PDMS layer under mask aligner. This is a critical step as any misalignment of the coplanar electrode can substantially reduce the sensitivity to sense the cells within the sensing zone. SI Fig. 4a shows the perfect alignment of microelectrodes with microfluidic channels as required in the designed layouts. SI Fig. 4b depicts the practical alignment as obtained on the fabricated microelectrodes. Before bonding, holes are drilled into the PDMS for the microfluidic channel's inlet and outlets. A 0.4 mm BD precision glide needle is used to punch holes, as shown in SI Fig. 4c.

**Figure 4:**
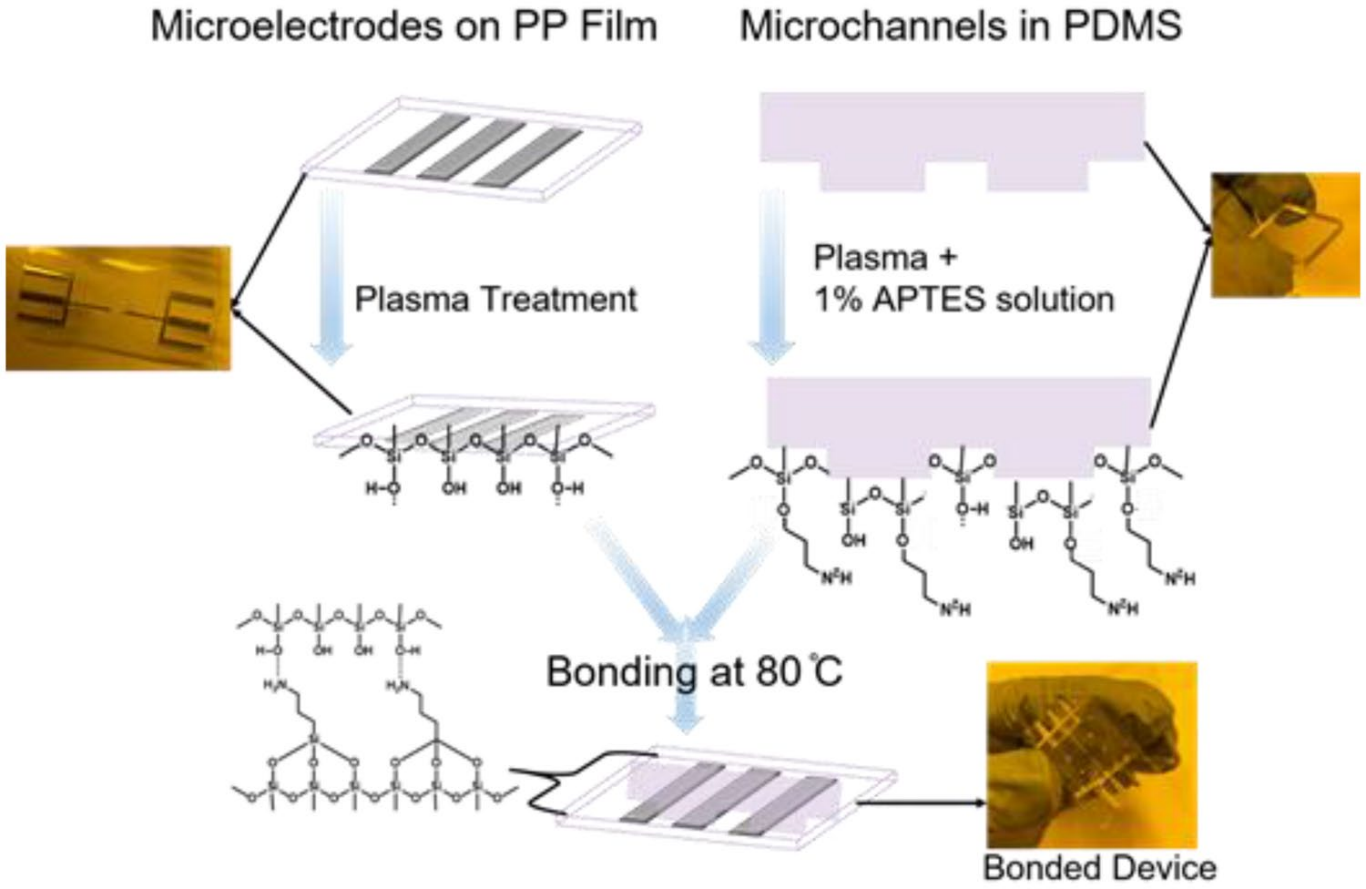
Schematic of the bonding process. An irreversible bonding is formed between microelectrodes patterned polypropylene (PP) film, and microchannels defined PDMS.

When the surfaces are exposed to the ambient conditions, poor bonding can occur due to crosslinking between the amino and hydroxyl groups on the surfaces. In some cases, a strong bonding is only possible within a few seconds of the surface treatment. After bonding, we anneal the device at elevated temperatures (80–100 degrees Celsius for 2–4 h) and under ~ 1 kg weight to prevent the inward diffusion of water at the bonded interface^58,59^, and to activate stronger bonds as illustrated in Fig. 4.

### Experimental setup

**Figure 5:**
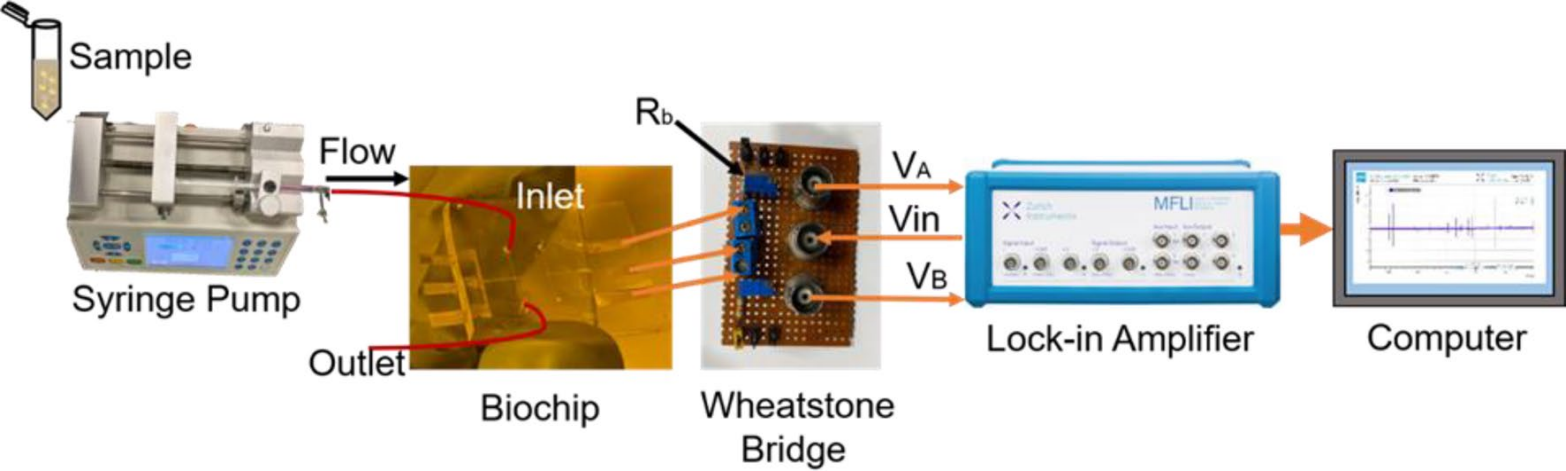
The experimental setup schematic represents syringe pump, flexible biochip, Wheatstone bridge, lock-in amplifier, and computer-aided software for data analysis.

The three extended ends of the electrode (1–3) are connected to a small Wheatstone bridge set up on a Vero board using conducting epoxy for simple and facile electrical output (SI Fig. S5 & Fig. 5). An input sinusoidal signal of 1 V (rms) at 300 kHz frequency is provided to the biochip central electrode using a Zurich instrument MFLI lock-in amplifier. To enable a smooth flow of cells/particles through the sensing zone, a syringe pump with a flow rate of 0.1 µL/min is used at the inlet. The cell flow is monitored under the inverted microscope.

The electrical potential drop across a resistor of 47 kΩ (R_b_) in the Wheatstone bridge setup is used to calculate the impedance change (ΔZ = Z_A_–Z_B_) across the electrodes when a particle passes through the detection zone^60^. Moreover, a bipolar pulse is obtained from the differential signal V_A_–V_B_, that represents the footprints of a particle or cell. It can further be translated into current or amplified as required.

The resulting amplified signal amplitude versus time is displayed on a lock-in amplifier plotter. The data is recorded at a specified (~ 13.3 kSa/sec) sampling rate, about 10 times higher than low pass filter bandwidth, automatic bandwidth control for noise reduction in MFLI instrument. Data analysis is done using programmed script written in MATLAB. The pulse count, maximum amplitude, and width are calculated at specified initial settings (~ frequency, voltage, etc.).

## Results and discussion

### Physical characterization

**Figure 6:**
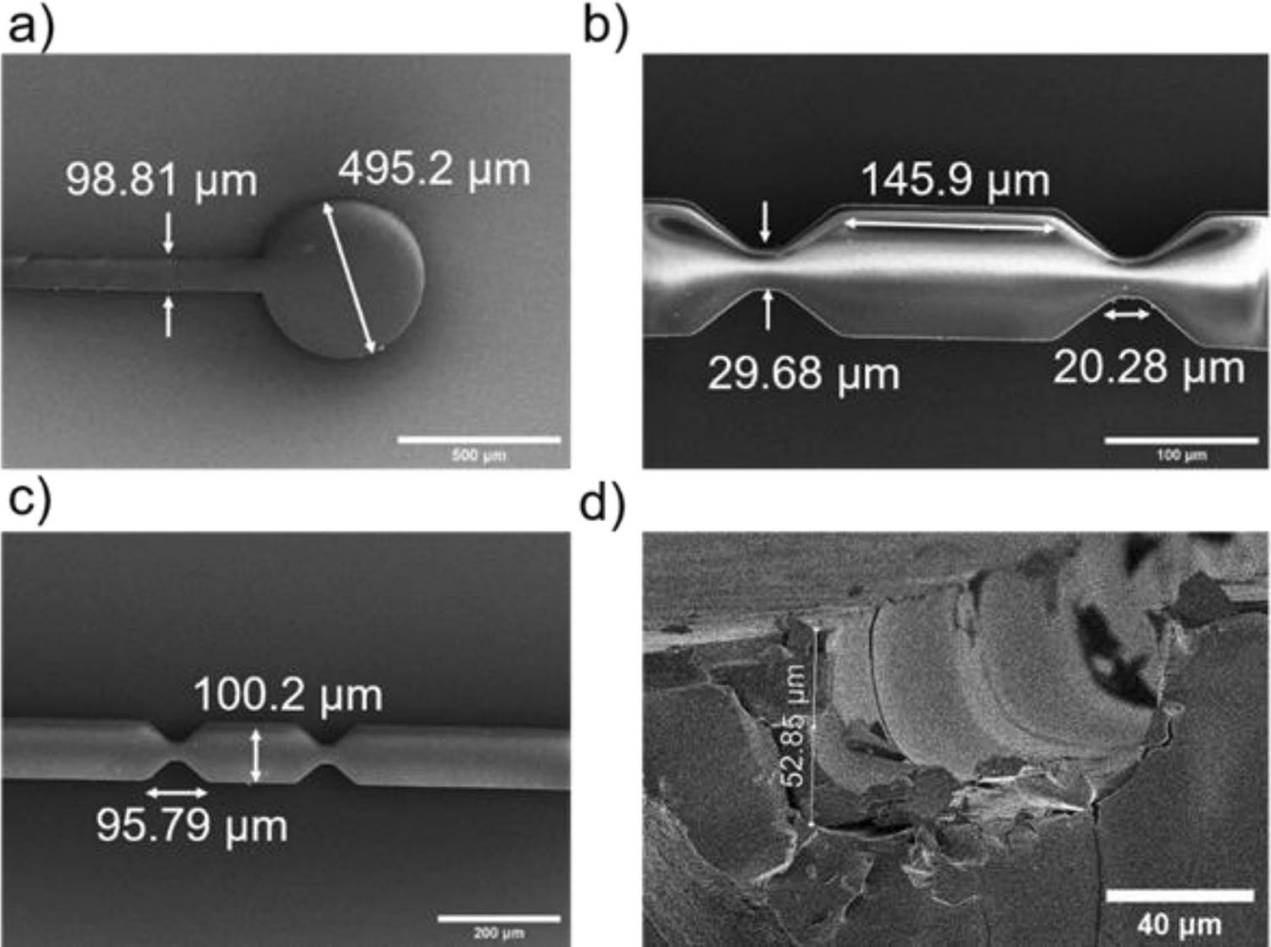
SEM micrographs of microfluidic channel fabricated in SU-8 after photolithography. (**a**) The inlet and outlet holes diameter of 500 µm and 100 µm microfluidic channel width, (**b**,**c**) Constricted sensing zone developed in SU-8 with comparable dimensions as defined in photomask, (**d**) Cross-sectional SEM image of PDMS microchannel created from SU-8 master stamp after soft lithography method.

Each phase of the process has a significant impact on the result and must be customized to the specific application. Figure 6a–d shows the 50 µm thick SU-8 microstructures fabricated by optimal UV lithography exposure and baking temperature and time. The structure contour is well defined at the constriction region in the middle. The minimum feature size of the structures in Fig. 6 is 20 µm with an aspect ratio of 2.5. Figure 6a shows an SEM image of a microchannel inlet/outlet with a diameter of 495.2 µm. The microchannel width is around 100 µm as compared to the sensing region width of 30 µm. The constricted channel length is up to 20 µm on both sides of the middle broad channel region. A well-defined tilted sidewall can be observed in the constricted area, joining the sensing zone with a wide microchannel. Figure 6d is the cross-sectional SEM image of PDMS microchannel fabricated through soft lithography methods using SU-8 patterns on silicon master stamp. The designs are well translated with similar height and tunnel shape microchannel defined within PDMS.

**Figure 7:**
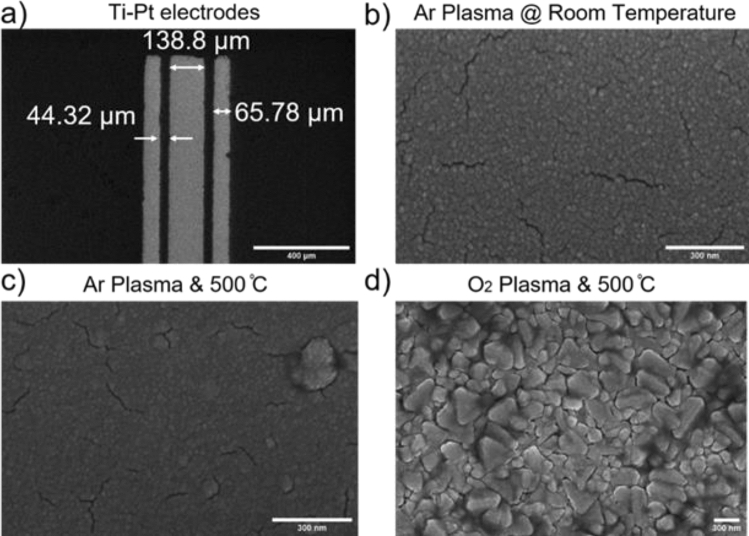
SEM images of microelectrodes fabricated on a polypropylene film. (**a**) Three coplanar electrodes with dimensions according to photomask Design M, (**b**) Titanium and platinum thin film deposition in argon plasma at room temperature, 40 nm & 100 nm, respectively, (**c**) Ti/Pt thin film deposited in argon plasma in magnetron sputter at elevated temperature, 500 °C, (**d**) Ti/Pt thin film deposited in oxygen plasma in magnetron sputter at elevated temperature, 500 °C.

We investigate various growth parameters (film thickness, growth temperature, atmosphere, chamber vacuum) to optimize the Pt/Ti thin film deposition for microelectrodes. The temperature and atmosphere effects are shown in Fig. 7. The detailed magnetron sputter deposition conditions are given in the SI Table S3.

The Pt film of 100 nm is grown on the 30 nm titanium layer in the same deposition conditions. The titanium film served as an adhesion layer between platinum and substrate (Glass or PP). To observe the effect of growth conditions on platinum film, it was first deposited directly on the substrate and film structure analyzed by XRD diffractometer using Cu Kα radiation. SI Fig. S5 shows the XRD pattern of platinum film of 100 nm thickness deposited at room temperature. From the XRD data the crystallite (grain) size of Pt/Ti thin films is about 18.42 nm as defined in the Scherrer Equation^61^. The peak intensity for Pt (111) is high as compared to other peaks, Pt (200) and Pt (220). It shows that deposited film is highly Pt (111) oriented, as detailed in various articles with comparable results^62,63^. The effect of deposition temperature and environment on 100 nm platinum film grown on the underlying adhesion layer of titanium (~ 30 nm) are displayed in Fig. 7. The surface grain size increases at elevated temperature with more cracks^64^. High temperature leads to the development of many structural defects and the production of the non-uniform film surface. There might be the oxidation of the titanium adhesion layer by reaction with oxygen in the chamber at elevated temperature (Fig. 7c,d). This issue is eliminated using inert conditions (i.e., Ar flow) at room temperature for the deposition of both thin films, i.e., titanium, and platinum (Fig. 7b). After a successful liftoff process, the optimized Ti/Pt thin film microelectrodes with listed parameters in SI Table S3 is obtained as shown in Fig. 7a. In SI Fig. S6, a few out of many adherence problems during optimization of platinum and titanium thin film growth parameters are displayed. We adopt a simpler approach to assess the adhesion quality using scotch tape-based method (see SI Fig. S6). Based on the adhesion test, the thin film deposition parameters are optimized. Although a simple adhesion test was considered sufficient for the process optimization, we should mention that more quantitative approaches such as conductivity measurements could be used in future for detailed analysis.

The surface treatment changes the film, glass, and PDMS surface properties, especially their hydrophobicity. The surface becomes hydrophilic after effective treatment. The prime test entails placing a water droplet (approximately 30 µl) on each surface and measuring the contact angle. A contact angle of fewer than 20 degrees would typically result in good bonding power. Owing to the presence of slightly more hydrophobic methyl groups in them, the hydrophobicity of pristine PDMS and PP is higher than that of glass. As compared to PDMS and glass, the rate of surface conversion for PP is much faster. SI Table S1 shows the water contact angle for surface modified PP and PDMS using various techniques. After the UVO treatment for 30 min, the water contact angles decreased to 60–85 degree^65,66^; however, air plasma treatment increases hydrophilicity down to 40 °, regardless of the substrate types their original surface wettability. Similar effect has been reported in literature for PP film^67–74^. After plasma treatment for 10 s, the contact angle decreases to 60°^67,68^. The air plasma probably breaks the C–C and/or C–H bonds to introduce C–O, C=O, and O–C=O groups. The contact angle changes from 111.48 to 70 for PP film after air plasma treatment for 150 s at different powers (0 to 190 W)^69^. K. N. Pandiyaraj et al. reported that pristine PP contact angle of 98.3̊ and air plasma treatment for 20 s decreases the angle to 49.4^70^. The O_2_ plasma treatment brings the water contact angle down to 17.8 for PP^71,72^ and less than 30 for PDMS and glass. After oxygen plasma and APTES salinization, the surface becomes completely hydrophilic (WCA < 20°)^73,74^, the water contact angles decrease substantially compared to those in the air plasma, and UVO treated surfaces, and the increment is generally greater for oxygen plasma than for others. The introduction of polar groups and the deposition of charged functionalities may cause the decreased water contact angles after treatments with UVO, air, and O_2_ plasma, with and without APTES, compared to those on pristine substrates. The water contact angle measured immediately after APTES treatment for 20 min is almost like the initial values^75^. APTES salinization helps substrate sealing by making irreversible bonds with hydroxyl groups present on the opposite surface by hydrogen bonding and allows sufficient time for the electrodes’ alignment with the microfluidic channels without affecting the strength of the bonding. The oxygen plasma without APTES treatment may lead to weak bonding if time delay increases above 10 min^76^. Hence oxygen plasma treatment combined with APTES salinization facilitates a comfortable time for the alignment step and yields irreversible chip bonding^77^ providing leakage-free flow around the inlet, outlet and at the channel edges.

The bonding strength is determined by the high flow rate sustainability of PDMS bonded microchannels with the substrate (SI Table S2). Injecting liquid inside microchannels at a high flow rate through syringe pump can cause PDMS separated from the substrate, leakages around the inlet or outlet holes or at the edges of the channel. With O_2_ plasma + APTES treatment, no leakage is observed at a high flow rate confirming that the bonding is strong & irreversible. The Fig. S7 highlights weak bonding issues during optimizing parameters for chip bonding and sealing. These optimized bonding techniques can be employed in the development of flexible biochips for applications in dynamic environments. Flexible biosensors have many appealing applications due to their lightweight, ultra-conformability (bendable, foldable, stretchable), compactness, disposability, and affordability. For instance, a cell counting device, the flexibility can enable use for wearable applications. For example, a wearable complete blood counter can be made that could be easily integrated with the human body. Supplementary Table S4 enlisted reported articles using polypropylene (PP) film as a flexible substrate in various applications.

### Cell counting

Polystyrene beads of 10-µm diameter are suspended in D1-water and sonicated to separate the clumped particles. Leukemia isolated cells are cultured in Roswell Park Memorial Medium (RPMI) and incubated at 37 °C in 5% CO_2_. To validate cell growth and their count per mL, a 15 µl cell suspended solution is injected in a glass-made Hemocytometer chamber with a cover slip at the top. An inverted microscope is used to count the cell numbers per mL. Ten microliters of cell suspended fluid is infused at the inlet port at 0.1 µL/min via syringe pump, as shown in Fig. 5. The lymphoblast cells are electrically counted as they pass through the constricted channel aligned with coplanar electrodes.

The microscopic image of aligned and bonded microchannels with microelectrodes for Design M & S is displayed in SI Fig. S3b,e, respectively. Three platinum electrodes (1–3) connected with the electronic measurement setup used to count individual cells are presented schematically in SI Fig. S8. The input voltage of 1 V (peak-to-peak) is fed at 300 kHz to the middle electrode from the lock-in amplifier output signal. The measured amplitude is a good representation of the cell size passing through biochip^78–80^. Due to cell passage between electrodes, the impedance change is recorded as a unipolar pulse ~ V_A_, and V_B_ via Wheatstone bridge circuit with resistances, R_b_ of 47 kΩ^81^. Both pulses (V_A_ and V_B_) are fed to the input of the lock-in amplifier, which takes the differential to remove the common mode noise between them and generate a bipolar pulse for each cell passing through the electrode on the plotter (see Fig. 5). The amplitude of the bipolar pulses is the electronic signature of the cell size used as a counting and differentiation technique.

**Figure 8:**
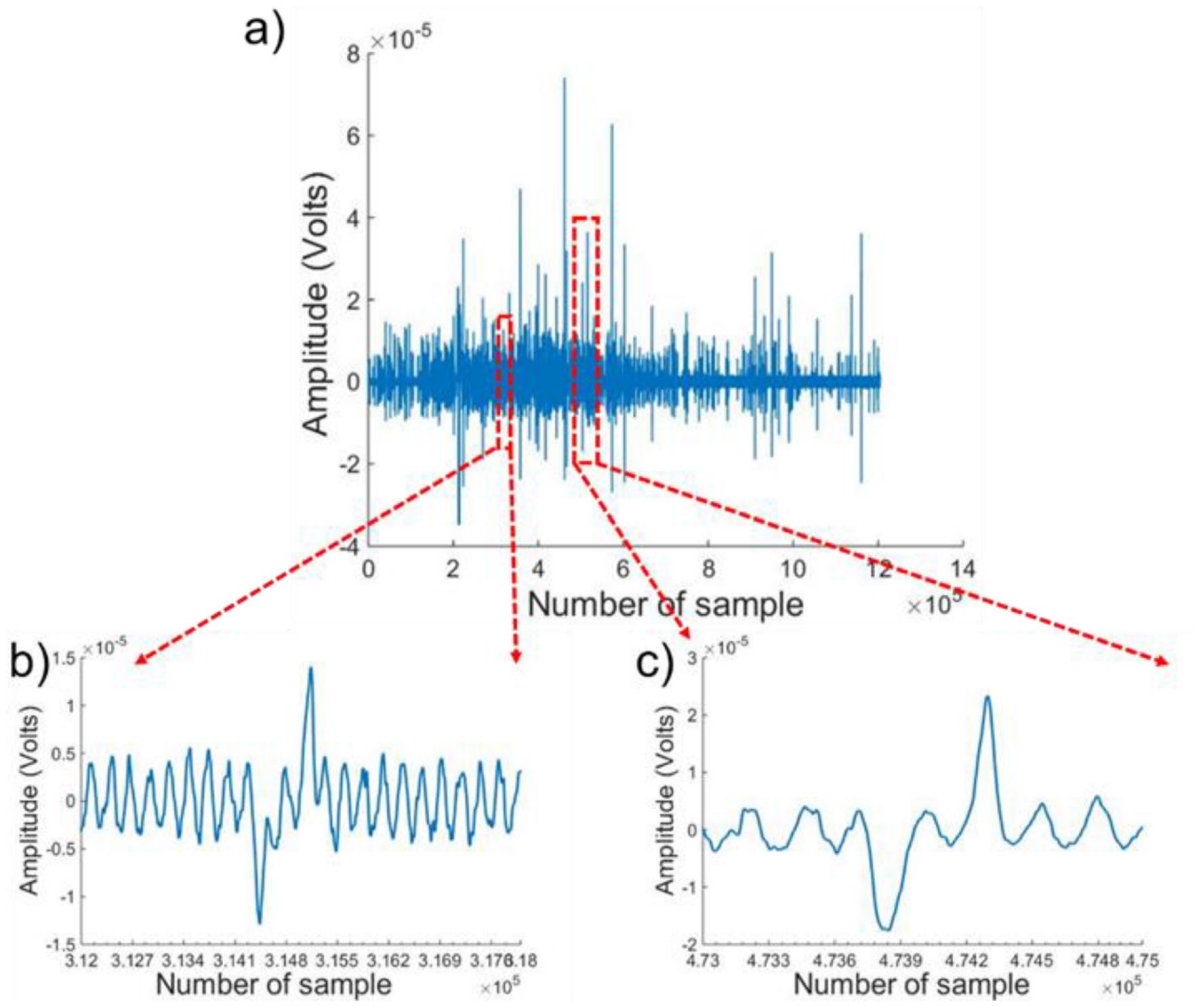
Bipolar pulses amplitude measured for the translocation of the leukemia cells through the microchannel. (**a**) Pulse’s amplitude recorded for a heterogeneous population of leukemia cells for 1200 s at 0.1 µL per min flow rate at sampling rate of 1.67kSa/sec, (**b**) A magnified bipolar pulse with smaller amplitude recorded, (**c**) A zoomed-in bipolar pulse for a large size leukemia cell passing through the microchannel.

A typical bipolar amplitude signal for a heterogeneous leukemia cell population (MV4-11) is shown in Fig. 8 which shows the voltage signal acquired for 1200 s during the sample flow. Voltage pulses with different amplitudes represent different sizes of cells in leukemia cell culture. The counted pulses peak amplitude is segregated and accountable at 2.5 times the standard deviation of the background noise. Figure 8b,c show the voltage amplitudes for bigger and smaller cells respectively. The average diameter range of lymphoblasts is 6–21 µm^82–84^.

**Figure 9:**
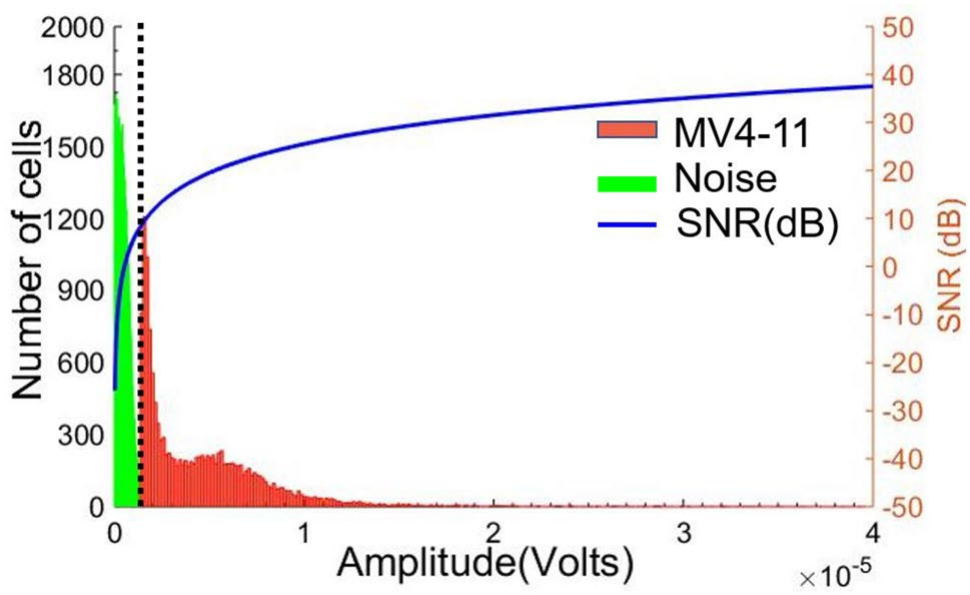
Histogram of pulse peak amplitude. The black dotted line separates the noise region on the left side of the histogram from the regular cell pulses amplitude. The signal-to-noise ratio is plotted on the same histogram by calculating the root-mean-square (rms) value of the pulse amplitude and the standard deviation of noise for 50,000 samples.

The histogram for the peak pulse amplitudes for the cell flow experiment is shown in Fig. 9. The dotted line separates the background noise from the pulse's amplitude. The cell count is supposed to adopt a uniform distribution throughout the experiment. Figure 9 is the histogram of the heterogenous size leukemia cell population with macrophage morphology suspended in fluid. The amplitude distribution represents the cell size variation within freshly prepared leukemia grown culture. The calculated pulse data average value is about 5.86 µV and a standard deviation of 4.4 µV. Signal-to-noise (SNR) is measured by finding the root-mean-square (rms) value of the pulse's amplitude and the standard deviation of 50,000 samples of noise. It is calculated and plotted for the whole range of data, as shown in Fig. 9. Comparing the presented biochip electrical count rate with the standard laboratory counting tool (hemocytometer). The hemocytometer count rate which was measured just before starting the biochip flow experiment is 9.75 × 10^5^ cells per mL. In comparison, the cell counter biochip counted 9.08 × 10^5^ cells per mL, which closely matches with the hemocytometer count. As hemocytometer counting method can suffer from the human error of over counting and mishandling, the electrical biochip counter enables automatic, faster and more reliable cell counting.

**Figure 10:**
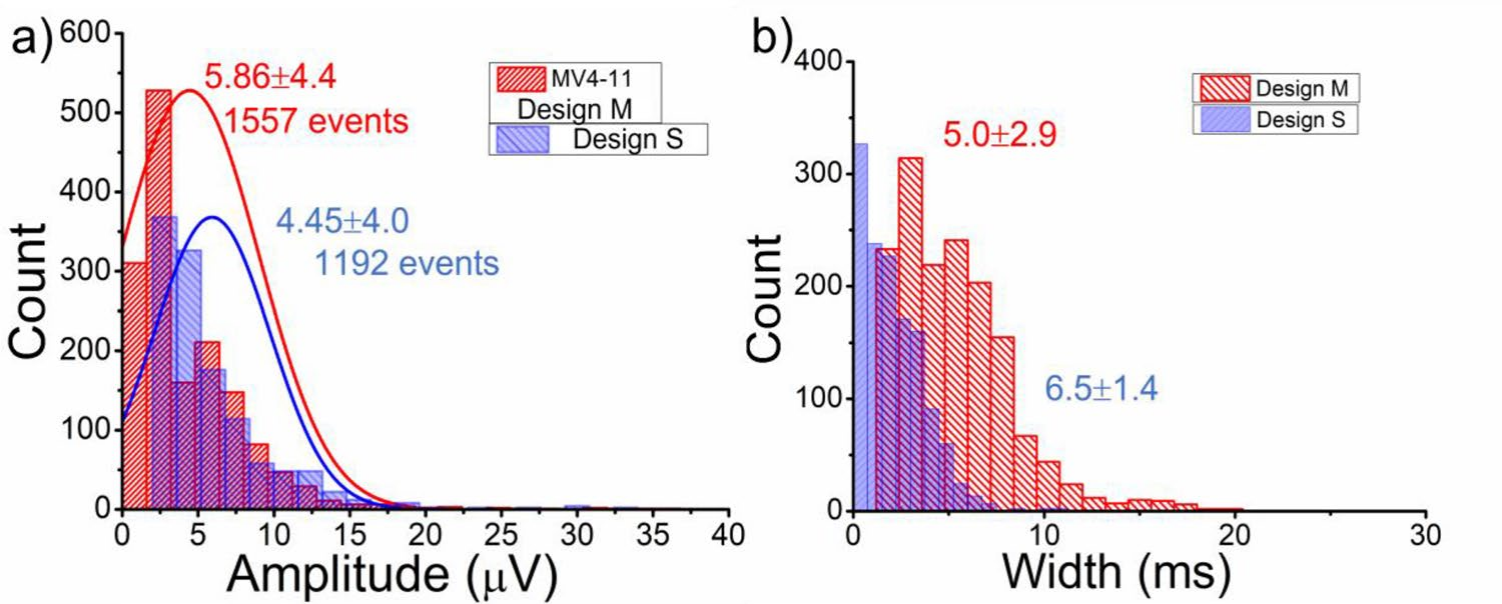
Pulse amplitude and width histograms for standard and modified electrode design (Design S & M) with two colors are shown (the number of events indicated in each case). (**a**) A comparison amplitude histogram plot for Leukemia cells passing through Design S and Design M biochips. The amplitude increment in the mean value for the presented design is observed at 300 kHz input voltage frequency, (**b**) Comparative histogram for pulses width indicates reduction compared to Design S due to the small detection region.

To further validate the performance of the fabricated biochip, a suspension of 10 µm diameter polystyrene beads (Sigma Aldrich) in PBS at 6.78 × 10^5^ beads/mL is prepared and then perfused at 0.1 µL/min flow rate. The experimental data recorded is shown in SI Figs. S9 & S10 with zoomed-in pulse and amplitude histogram with noise and signal-to-noise ratio (SNR). In this present study, we have used coplanar configuration with three electrodes set up with a modified Design M consisting of a 370 µm cell detection region and compared with standard Design S with 500 µm cell detection region (see SI Fig. S1a,b). Reducing the detection region length decreases the pulse width as compared to previously reported articles^85^. Interestingly, however, the positioning of the two outer electrodes under the constricted sensing region of the microchannel (in Design M) increases the overall pulse amplitude with an enhanced signal-to-noise ratio (~ max. SNR = 38.78 dB). Comparing histograms for pulses’ amplitude and that for the pulse width of the new electrode Design M with the standard coplanar electrode Design S are displayed in Fig. 10.

The Design M provides a higher signal strength with an average peak amplitude of 5.86 µV, as the cell passes between the electrodes as compared to the Design S with a mean amplitude value of around 4.4 µV. The Design S provides a broader signal with average pulse width is 6.5 ms because of a longer passing time between the electrodes. To evaluate the performance of each electrode design, an electrical cell measurement is performed at a range of frequencies 100 kHz, 500 kHz, 700 kHz, 900 kHz, 1 MHz, 3 & 5 MHz. The comparison plots for both designs are displayed in SI Figs. S11 & S12, and values are tabulated in SI Tables S5 & S6. At lower frequency ≤ 100 kHz, the background noise contribution is higher than the actual cell signal. While a distinguishable bipolar pulse corresponding to cell volume is detectable at frequencies ≤ 300 kHz and ≥ 900 kHz. But higher signal to noise ratio is achievable at 300 kHz for the Design M with cell count matches with the standard. While in case of Design S higher SNR is obtained at 700 & 900 kHz but enormous amount of signal peaks is undetectable. Hence best frequency fit for cell signal detection corresponding to cell size for both designs is chosen to be 300 kHz.

**Figure 11:**
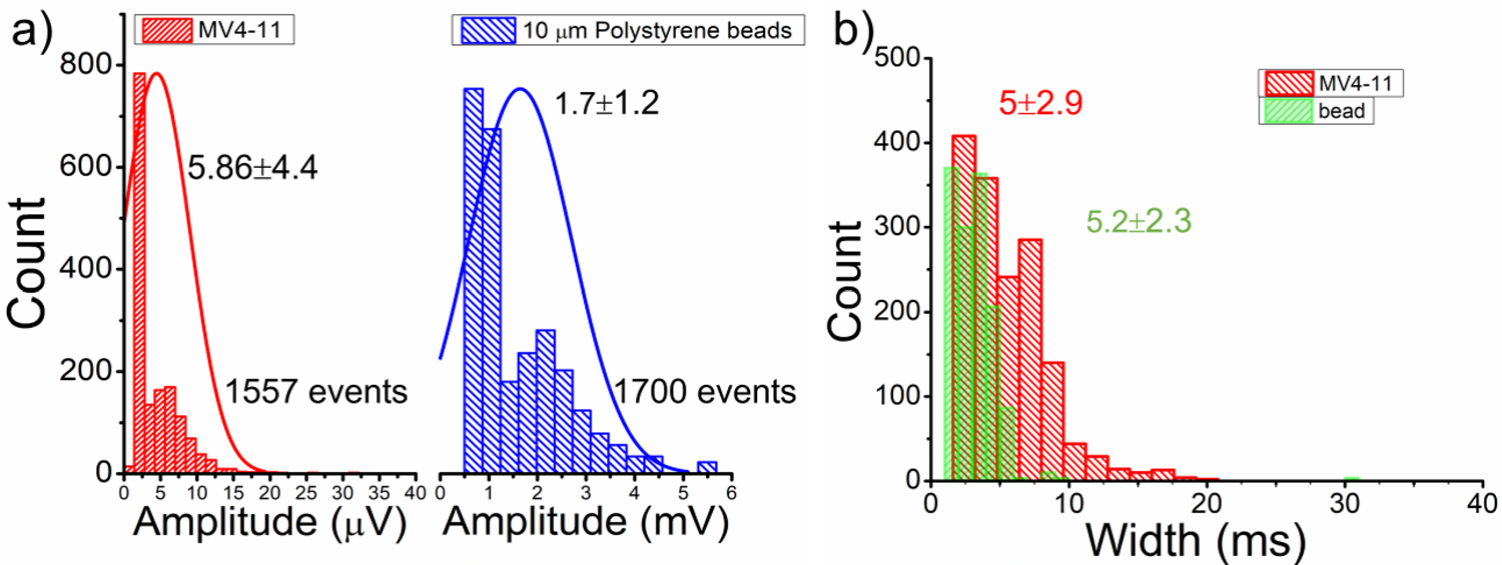
Histogram for the spike amplitude and pulse width for a heterogeneous population of leukemia cells and 10 µm polystyrene beads for 800 s recorded data with several events with curves shows normally distributed fitting results.

The pulse amplitude and width histograms for leukemia cells and beads in Design M biochip are compared in Fig. 11. The amplitude and pulse width for both particles show fitted curves for the normal distribution. The average amplitude for leukemia cells is 5.86 µV with a 4.4 µV standard deviation. While for beads, the average amplitude calculated is 1.7 mV with a standard deviation of 1.2 mV. The pulse width average and standard deviation for leukemia cells are 5 ms and 2.9 ms. Bead pulse width average lies in the range of 5.2 ms and standard deviation of 2.3 ms. The width of the pulses doesn't demonstrate much difference in their histogram. Pulse amplitude, width, signal to noise (SNR) at maximum and average point are listed in detail in SI Table S7. The leukemia cells have a wide range of cell diameter (~ 6–21 µm), while beads have a 10 ± 2 µm diameter range. The biological cells are conductive as compared to polymer beads, which behave as an insulating sphere. Hence the electrical signature for bead has a higher amplitude recorded as compared to conductive leukemia cells. The pulse width is approximately the same, with a higher standard deviation in the leukemia heterogeneous cell population.

**Figure 12:**
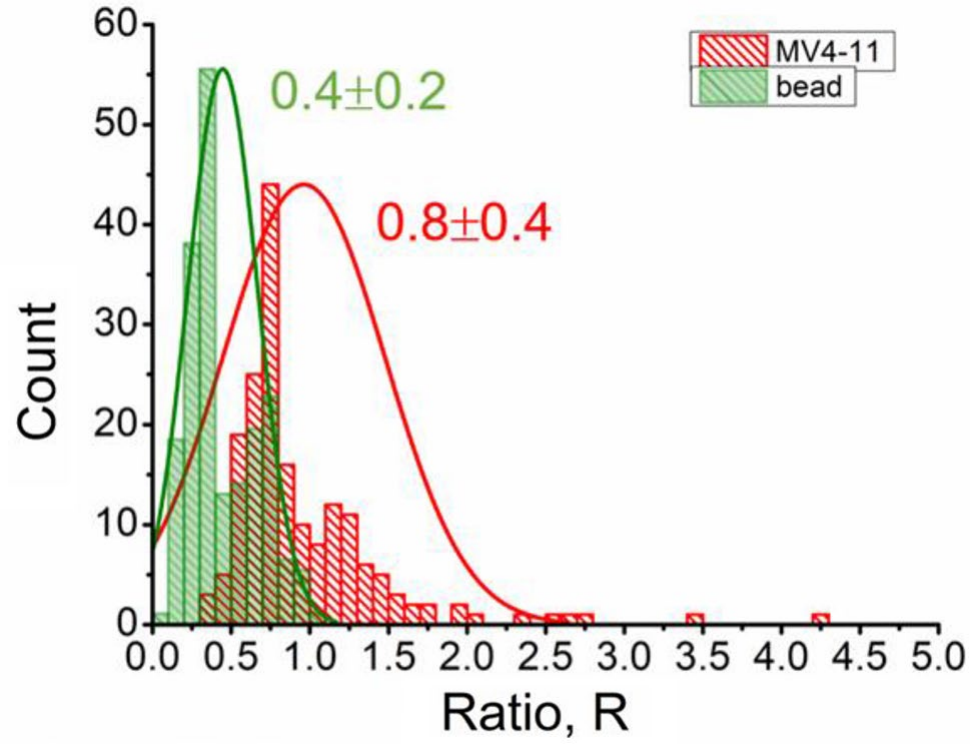
Two-color histogram of ratio, R of amplitude to pulse width for leukemia cells, and polystyrene beads with fitted normal distribution. Each histogram's average value and standard deviation are labeled.

To further investigate the sensitivity of biochips for particle type differentiation, a ratio, R, is defined as peak amplitude divided by the pulse width. The ratio describes different particles, such as biological cells or a polystyrene bead in our case. The calculated ratio, R of the amplitude to pulse width, gives a good understanding of particle type, as shown in Fig. 12. The R-value discriminates the type of particle passing through the electrodes^86^. The R-value showed normal distribution around 0.4 ± 0.2 and 0.8 ± 0.4 for the leukemia cells and beads, respectively. These results indicate that R-value could be used to discriminate between conductive leukemia cells and insulating beads. R-value can be used to differentiate a large population of heterogeneous particles of different types and sizes. The large size of leukemia cells has large volume occupied, and hence pulse amplitude and R-value are higher and discriminative compared to others. The average R-value for leukemia cells is around 0.8 shows that cell culture contains more leukemia cells with a diameter larger than 10 µm. For 10 µm diameter particle size, the R-value is about 0.4. When the amplitude is more significant than 1 mV and R is smaller than 0.5, the crossing particle is an insulating sphere.

**Figure 13:**
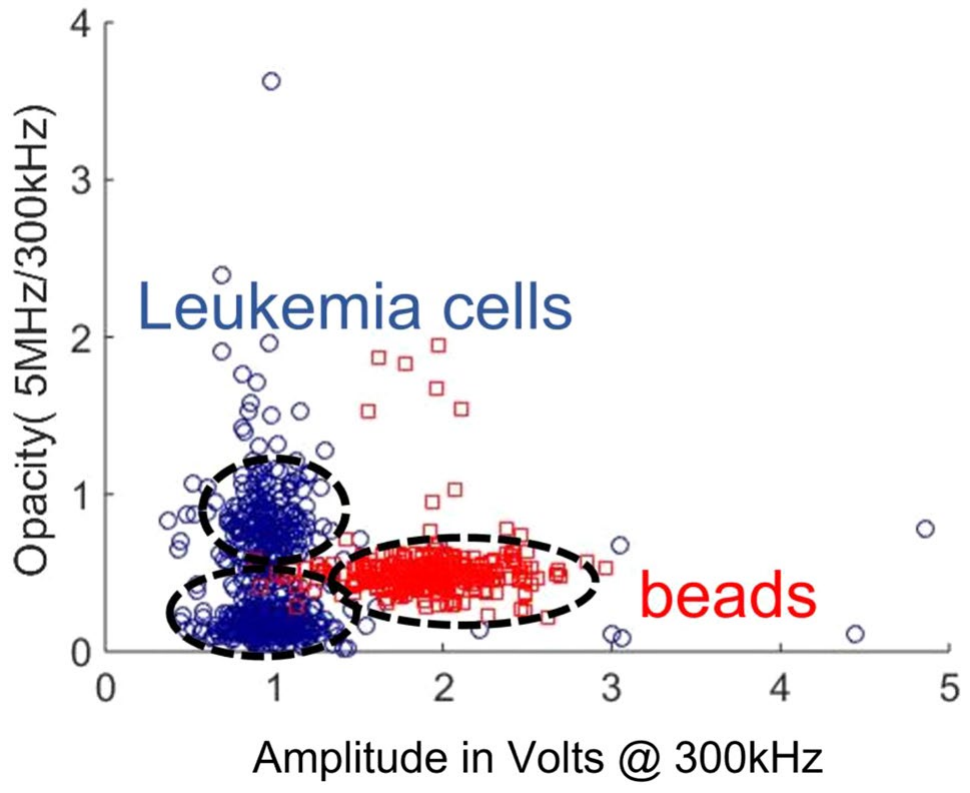
Opacity scatter plot of beads and leukemia cells at a low frequency of 300 kHz and a high frequency of 5 MHz.

The scatters plot for Design M biochip is presented in Fig. 13 to recognize better the morphological differentiation between leukemia cells and spherical beads. At the input frequency of 300 kHz, the cell passing through the sensing zone behaves as an insulating sphere, and the bipolar pulse obtained is the electrical signature of the cell size. Opacity is defined as a ratio of amplitude recorded at 5 MHz to the amplitude values at 300 kHz. The cytoplasm of biological cells is more conductive than polystyrene, giving decreased signal amplitude and reduced opacity. The scatter plot clearly shows the difference between insulating beads and leukemia cells. Similar trends are also observed in blood cell differentiation scatter plots in previous articles^87–90^. The two regions within leukemia scatter plot shows the heterogeneous population of leukemia cells in cell size and slightly variable interior cell properties within a cell culture.

The selectivity of the receptor-less cell counter is based on the particle size, morphology, and dielectric properties which can be directly applicable for counting red blood cells, white blood cells, and platelets in complete blood counting tests as shown in Hassan et al.^91^. Moreover, doing a multi-frequency impedimetric analysis similar to that shown in Fig. 13, sub-populations from a single type of cells, such as monocytes vs. lymphocytes within white blood cell population, can be selectively counted^92,93^. Another important application of receptor-less approach can be in the area of drug efficacy testing and pathogens detection^36,79,94^. An addition of a receptor layer can enable the cell counter to be useful in microfluidic point of care immunoassays that have been reported recently in literature^95–97^.

Finally, we should mention that although the scope of the presented work is lab-based research, many of the equipment and processes used in our method are routinely used in the microelectronics/semiconductor industry for the manufacturing of integrated circuits. Although some of the processes such as photolithography and thin film metal deposition using sputtering are expensive, the economic feasibility of this approach relies on the market size, demand, and a high capital investment. The economy of scale could work well if the market size is huge, and the return on investment could be attractive for the investors. The cost of a single biochip could be as low as that of an off-the-shelf microelectronic component (e.g., a flash USB drive) if the volume of production and market demand is huge.

## Conclusion

In conclusion, we explore design improvements in impedimetric microfluidic cytometer on flexible substrate through fabrication and characterization. We discuss process optimization for repeatable metal patterns of Ti/Pt thin films on polypropylene flexible layer and a systematic approach to enhance bonding strength between the PDMS and polypropylene. Experimental testing on polystyrene beads and a heterogeneous population of leukemia cells (MV4-11) having average diameters of 10 $\mathrm{\mu m}$ is carried out to evaluate biochips performance. An improved coplanar electrode layout shows enhancement in the sensor’s sensitivity as compared to the conventional design offering improvement in spike bipolar pulse amplitude of about 31.6%, reduced noise of about 15.6%, and a higher signal-to-noise ratio (SNR) of about 62.1%. The sensor’s accuracy is validated by comparing its output to the standard cell counting hemocytometry method. The selectivity of the device is analyzed by comparing the multi-frequency output for the polystyrene beads *vs.* leukemia cells. Flexible electrical biosensing devices are desirable for cost-effective and wearable point-of-care biochips for a broad range of biomedical and electronics applications. The methods outlined in this paper can be used to fabricate and characterize microfluidic cytometers for a broad range of applications.

## Supplementary Information

Supplementary Information.
