# Supplementary material for: Thin flexible lab-on-a-film for impedimetric sensing in biomedical applications
Source: Sci Rep. 2022 Jan 20;12:1066. doi: 10.1038/s41598-022-04917-5 (PMC8776742; doi:10.1038/s41598-022-04917-5)
Supplement: Supplementary file 1 — Supplementary Information. [file 41598_2022_4917_MOESM1_ESM.docx]

**Thin Flexible Lab-on-a-Film for Impedimetric Sensing in Biomedical Applications**

**Supplementary Information**

# Material List

**Reagents**

PBS, 1× (Thermo Fisher Scientific)

Acetone, deionized (DI) water, isopropanol (Laboratory-supplied chemicals)

AZ-1512 photoresist (MicroChemicals)

AZ-MIF 300 Remover (MicroChemicals)

SU8-100 photoresist (Gersteltec Sarl. Switzerland)

SU8 developer (MicroChemicals)

RPMI Media (Sigma-Aldrich)

MV4-11 cells (ATCCA)

Polystyrene beads (Sigma-Aldrich)

PDMS, Sylgard 184 silicone elastomer kit (Dow Corning, Ellsworth Adhesives)

Poly(propylene) (PP) thin films (20 μm thickness) Tri-Pack Films, Limited, Pakistan.

**Equipment**

Lock-in amplifier (Zurich Instruments, MFLI)

Inverted microscope (OLYMPUS-CKX41)

Syringe, 1, & 5 ml (Fisherbrand from Thermo Fisher Scientific, Lot no. 20191122c)

Puncher (ROBBINS INSTRUMENTS, Lot no. 16343)

BD precision tips, 10, 200, and 1,000 µl (Thermo Fisher Scientific)

Glass Slides (Premiere, cat.no. 6101)

Microbore polytetrafluoroethylene (PTFE) tubing (JMS Singapore PTE, LTD)

Ultramicrobore PTFE tubing (Cole-Parmer)

Hot plate (CHEMAT, Technology, INC.)

Conductive silver epoxy (Ted Pella Inc. Product no. 16043)

A syringe pump (Chemyx, Inc. Model: Fusion 100)

**Software**

AutoCAD (to design micrometer features of electrodes and channel for photomasks)

ZI Control (to operate lock-in amplifier: https://www.zhinst.com/ products/mfli)

MATLAB (to analyze the collected data: https://www.mathworks.com/ downloads/)


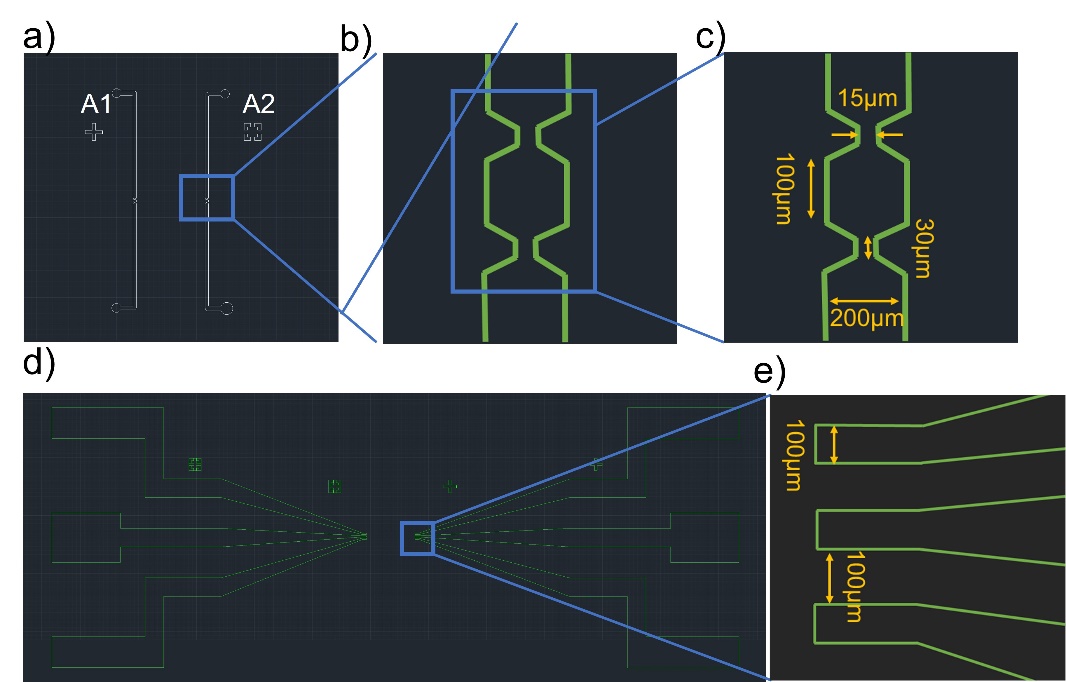

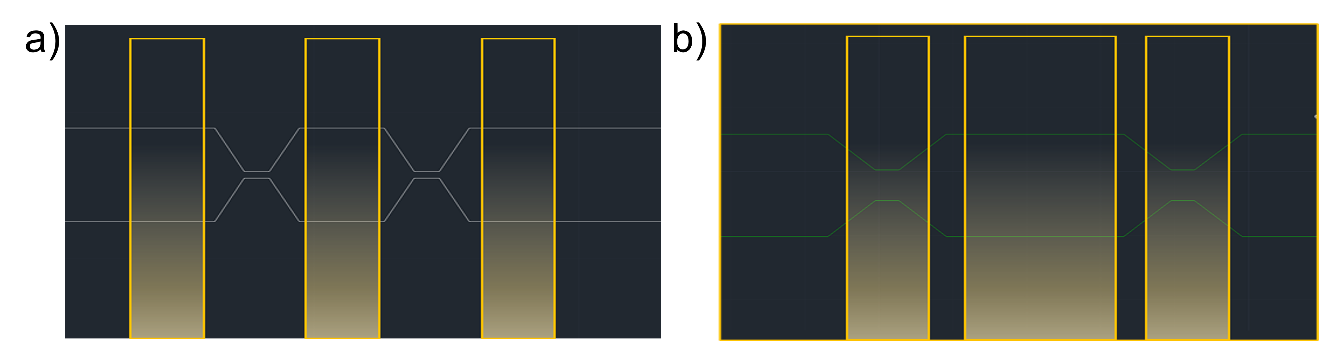


Fig. S1 a) Coplanar three electrode layout aligned with constricted microchannel, a) Electrodes are placed at right and left side of both sensing regions in the standard (Design S) electrode configuration, b) while in proposed modified (Design M) configuration the peripheral electrodes are positioned under the sensing regions with the central electrode in the middle of them.

Fig. S2 Comparative standard microchannel and microelectrodes (Design S) layout on AutoCAD. a) single biochip microfluidic channels with alignment mark A1 and A2, b & c) The constricted channel region with dimensions, d & e) microelectrode design with zoomed-in dimensions of electrode region aligned with the sensing zone of a channel.


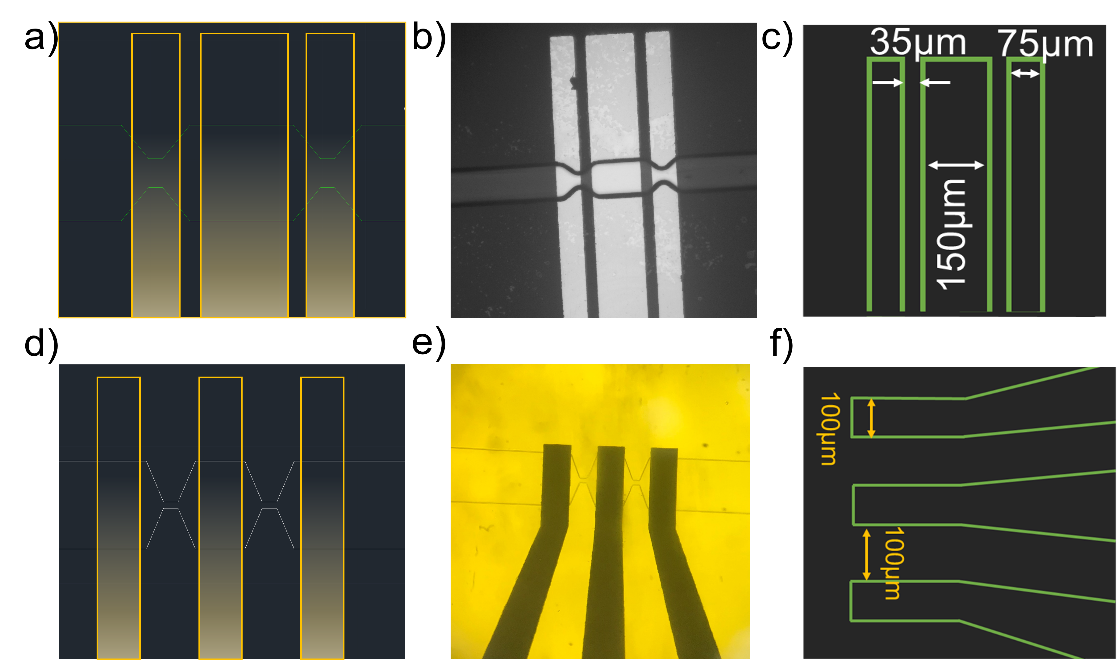


Figure S3. Standard and modified electrode design alignment with microchannel. a) Design M: Modified electrode design in AutoCAD aligned with microchannel design, b) Microscope image of new electrode design patterned on polypropylene film and aligned with PDMS microchannel, c) Dimensions of modified electrode design, d) Design S: Standard electrode design in AutoCAD aligned with microchannel design, e) Microscope image of standard electrode design patterned on polypropylene film and aligned with PDMS microchannel, f) Dimensions of standard electrode design.


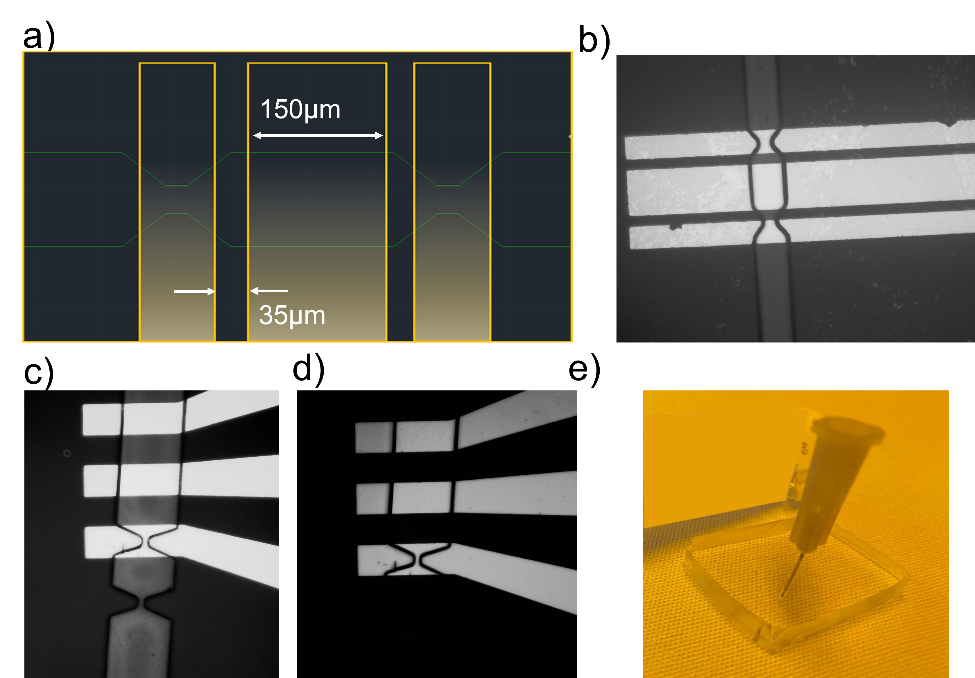


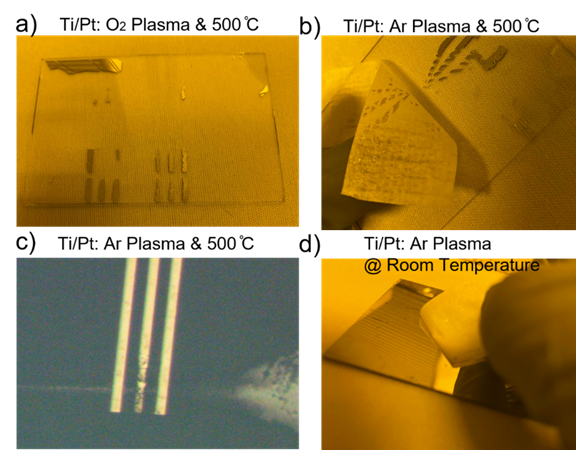

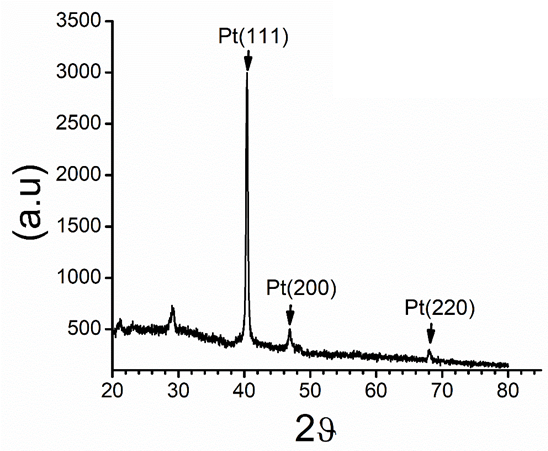


Fig. S4 a) Microelectrodes and microfluidic channels alignment in AutoCAD design for Design M, b) Aligned PDMS microchannels with microelectrodes on polypropylene film in mask aligner, c & d) misaligned electrodes examples, d) Inlet and outlet microchannel hole punching.

Fig. S6 Platinum and titanium thin film adhesion issues. a) Poor adhesion of Pt/Ti thin film resulted in complete wash off during liftoff process, b) Tape test to check the adhesion of Pt/Ti thin-film electrodes, c) Poor adhesion of Pt/Ti thin film electrodes resulted in parts of film removal when immersed in DI water, d) Excellent adhesion of both titanium and platinum films in the optimized magnetron sputter process parameters.

Fig. S5 XRD graph of Pt film of 100nm thickness deposited at room temperature in magnetron sputter.


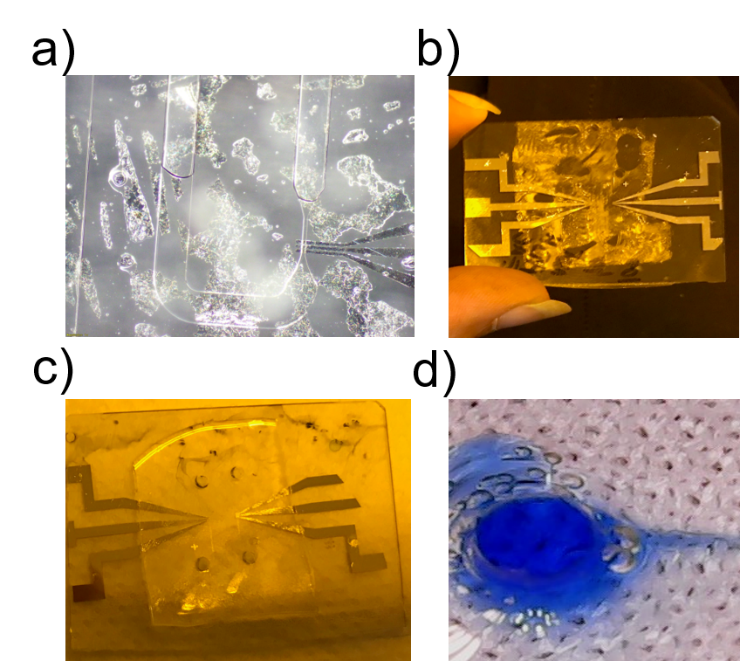


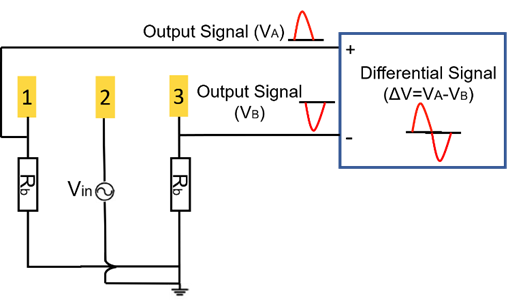

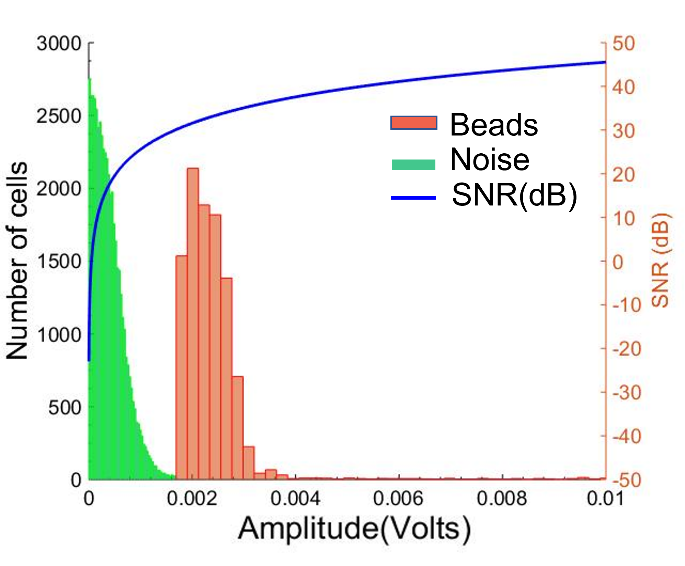

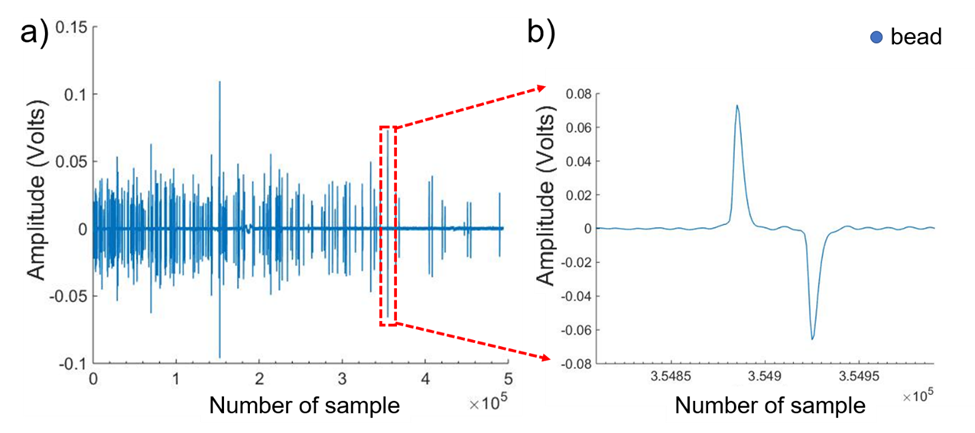


Fig. S8 A graphic representation of coulter cell counting set up for differential output signal measured as (ΔV=V_A_-V_B_) a potential drop across a resistor R_b_ in a Wheatstone bridge set up.

Fig. S7 **Bonding issues**. a) Poorly bonded PDMS with PP film, solution leaks all around the surface, b) bubbles formed during bonding leads to bonds breakage between two substrates, c & d) Weak bonding leads to leakage around inlet and outlet hole, spreads the test fluid all around the surface.

Fig. S10 a) Histogram of the spike amplitude versus the number of occurrences of the 10 µm polystyrene bead through the biochip. On the left side, the noise is plotted in green color, and amplitudes are in red. Signal to noise ratio (SNR) is also displayed in dB.

Fig.S9. a) 10 µm polystyrene beads bipolar pulses amplitude recording for 300-sec data during an experiment. b) Zoomed in single pulse representing single bead electrical signature recorded during impedimetric sensing.


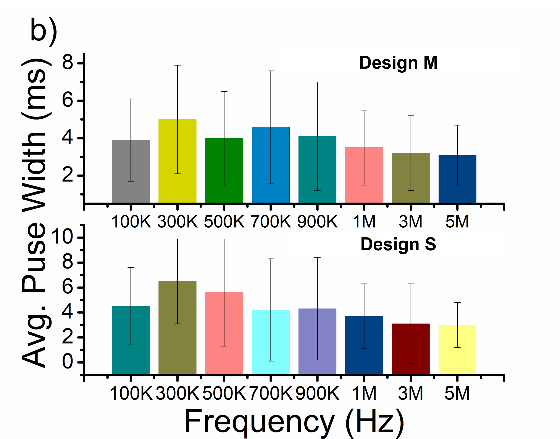


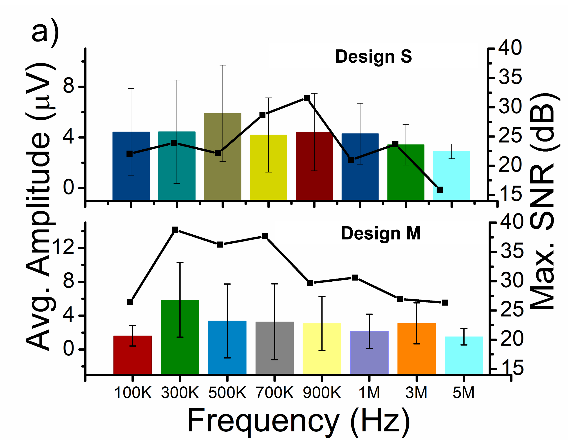


Fig. S11 a & b) Bar graph of the average value of leukemia cells pulse amplitude and pulse width at different input frequencies from 100 kHz, 500 kHz, 700 kHz, 900 kHz, 1 MHz, 3 MHz & 5 MHz in Design M as compared to Design S.


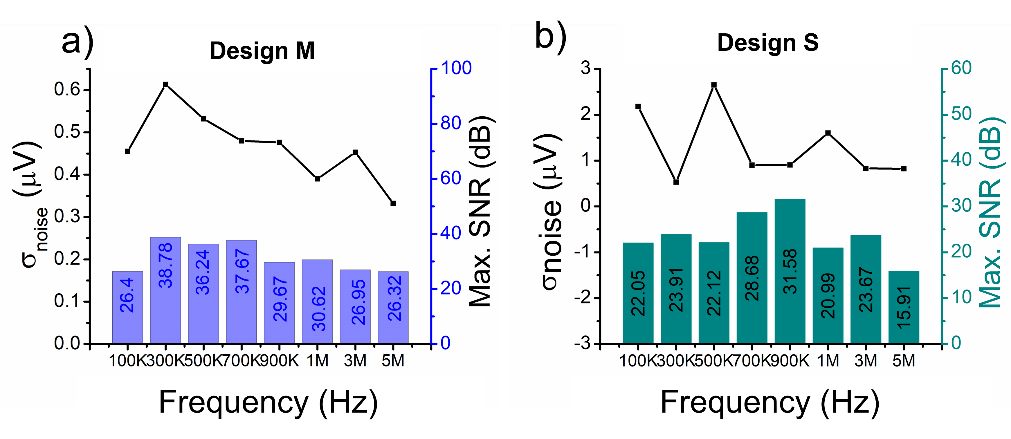


Fig. S12. a & b) Bar graph of standard deviation of noise in Design M as compared to Design S as leukemia cells passes through them at different input frequencies from 100 kHz, 500 kHz, 700 kHz, 900 kHz, 1 MHz, 3 MHz & 5 MHz. Maximum signal to noise ratio measured by the Design M biochip as compared to Design S biochip.

**Additional Information on cell counter’s mechanical distortion**

The mechanical distortion in flexible biosensors can result into performance variation. In wearable sensors, the bending of the device along the shape of the body can vary the signal output due to stretch, deformation, and distortion in the sensing region. For a significant mechanical distortion, the cross section of the sensitive volume can change which in turn may lead to changes in pressure distribution affecting the flow pattern and associated microfluidic functions. For the cell counter device presented in this paper, the sensitivity of the output signal is confined to an extremely small detection volume (20 um ×30 um × 50 um). We therefore expect that most common mechanical distortions (for example due to bending over the human body) may not have a significant change in the shape or size of the detection volume. Nevertheless, we plan to explore this effect in detail in our future research.

Table S1. Water contact angle measured on Pristine, UV, Oxygen/Air Plasma, and APTES treated Polypropylene (PP), PDMS, and Glass surfaces.

| **Material** | **Pristine** | **UV**  **(30-min)** | **Air Plasma**  **(10 min)** | **Air Plasma + APTES** | **O_2_ Plasma**  **(2 min)** | **APTES**  **(20 min)** | **O_2_ Plasma**  **+ APTES** |
| --- | --- | --- | --- | --- | --- | --- | --- |
| PP | 98.8 | 77.7 | 54.5 | 53.6 | 17.8 | 93.3 | 5.1 |
| PDMS | 109 | 84.2 | 64.6 | 62.1 | 26.3 | 95.8 | 9.5 |
| Glass | 89.7 | 62.1 | 43.3 | 58.6 | 24.2 | 84.2 | 7.1 |

Table S2. Effect of various surface treatments on bonding quality. Air plasma treatment is done at RF for 10-min, UV treatment is 30-minute long, Oxygen plasma is done at 60W for 2-minute, APTES solution is 1% v/v aq. at 60 C for 20-min. Strong bonding corresponds to zero leakage at high solution flow rate, PDMS is not peeled off from the substrate surface. Weak bonding indicates leakage at high flow rate, and × (No bonding) means PDMS peel off easily.

| **PP** | **PDMS** | **Bonding** | |
| --- | --- | --- | --- |
|  |  | Direct bonding  without any delay | Delay due to alignment (5-15min) |
| UV | UV | Strong | × |
| Air plasma | Air plasma | Weak | × |
| Air plasma | APTES | × | × |
| Air plasma | Air plasma  + APTES | Strong | Weak |
| O_2_-plasma | O_2_-plasma | Strong | Strong |
| O_2_-plasma | O_2_-plasma + APTES | Strong | Strong |

Table S3. Titanium and Platinum thin films magnetron sputter deposition parameters.

| **Deposition parameters** | **Ar Flow rate (sccm)** | **Base pressure (torr)** | **Voltage**  **(V)** | **Current**  **(mA)** | **Deposition Temperature**  **(̊C)** | **Target Rotation** | **Target to substrate distance (mm)** | **Thickness**  **(nm)** |
| --- | --- | --- | --- | --- | --- | --- | --- | --- |
| Ti | 60 | 9 × 10^-7^ | 300 | 50-60 | 23 | Yes | 70 | 30 |
| Pt | 60 | 9 × 10^-7^ | 360 | 40 | 23 | yes | 70 | 100 |

Table S4. Applications of Polypropylene (PP) film as a flexible substrate.

| Application | Technique | Membrane properties | Ref. |
| --- | --- | --- | --- |
| Amperometric creatinine biosensor | plasma-induced graft polymerization | PP membrane (1 mil thick) | [1] |
| Enzyme bioreactors | plasma-induced graft polymerization | lipase immobilized polypropylene microfiltration membranes | [2] |
| Viral gene isolation in bioseparation | chemical metal coating method | PP membrane | [3] |
| Cell separation | plasma-induced polymerization | adsorbed antibody for cell separation | [4] |
| Lectin Recognition | UV or plasma-induced graft polymerization | Glycosylated Surface on Polymer Membrane | [5] |
| Electrical detection of live bacterial cells | coated with a conductive polypyrrole and antibody functionalized | polypropylene microfiber membranes | [6] |
| Pseudo dual nucleic acid detection | laser engraving | polypropylene bag, double layers of PP film with thickness of 0.06 mm | [7] |
| Wearable biosensor for tear glucose measurement | “Soft-MEMS” | functional polymer membrane of 25 µm via amperometry | [8] |
| Wearable pressure (WP) sensors | cold plasma (O2) | Functionalization of polypropylene nonwoven fabrics | [9] |

Table S5. Average pulse amplitude, width, signal to noise ratio (SNR) at maximum & mean value, with several cells counted in 120 seconds by modified electrode design (Design M) biochip at different input frequencies.


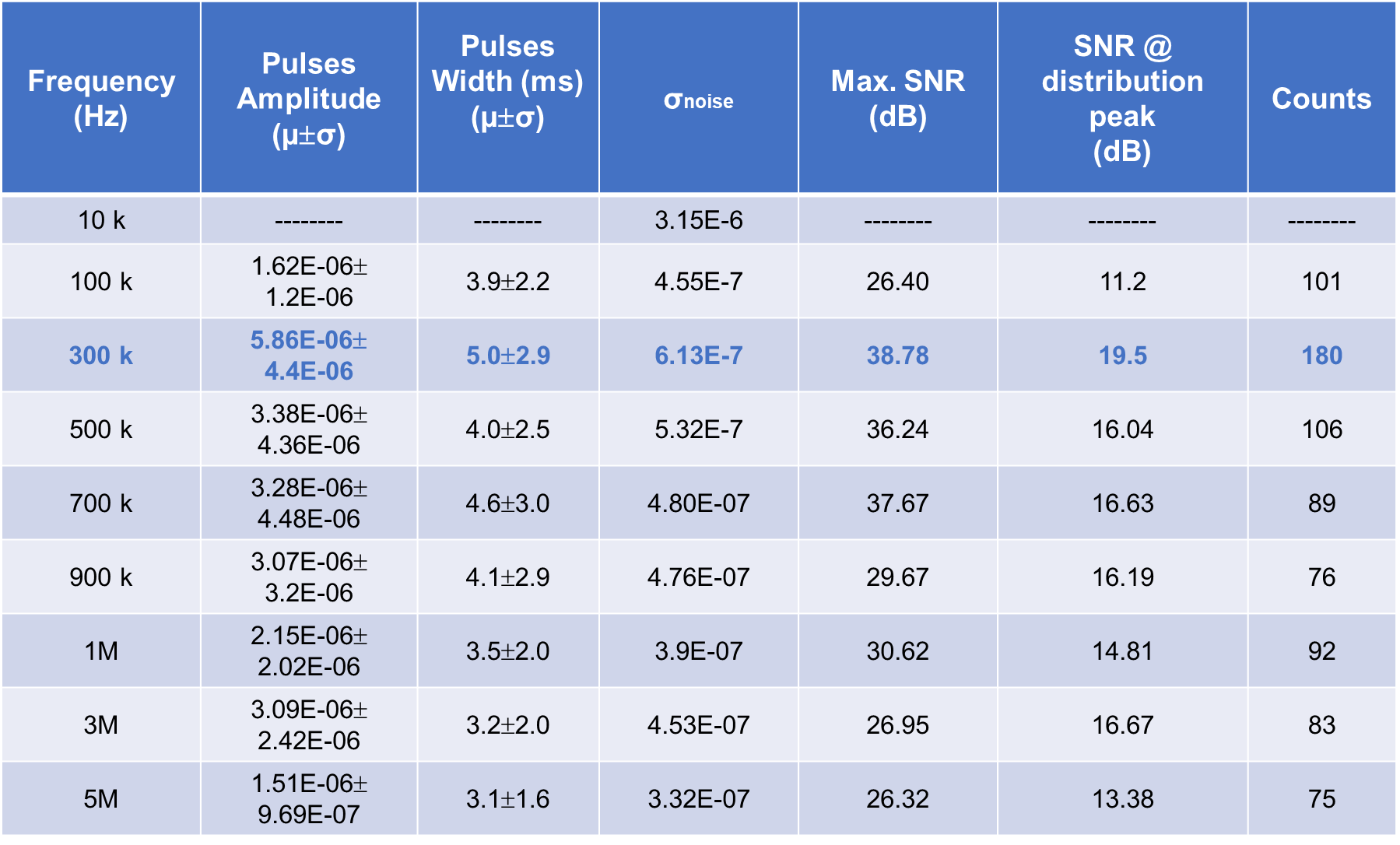


Table S6. Average pulse amplitude, width, signal to noise ratio (SNR) at maximum & mean value, with number of cells counted in 120 seconds by standard electrode design (Design S) biochip at different input frequencies.


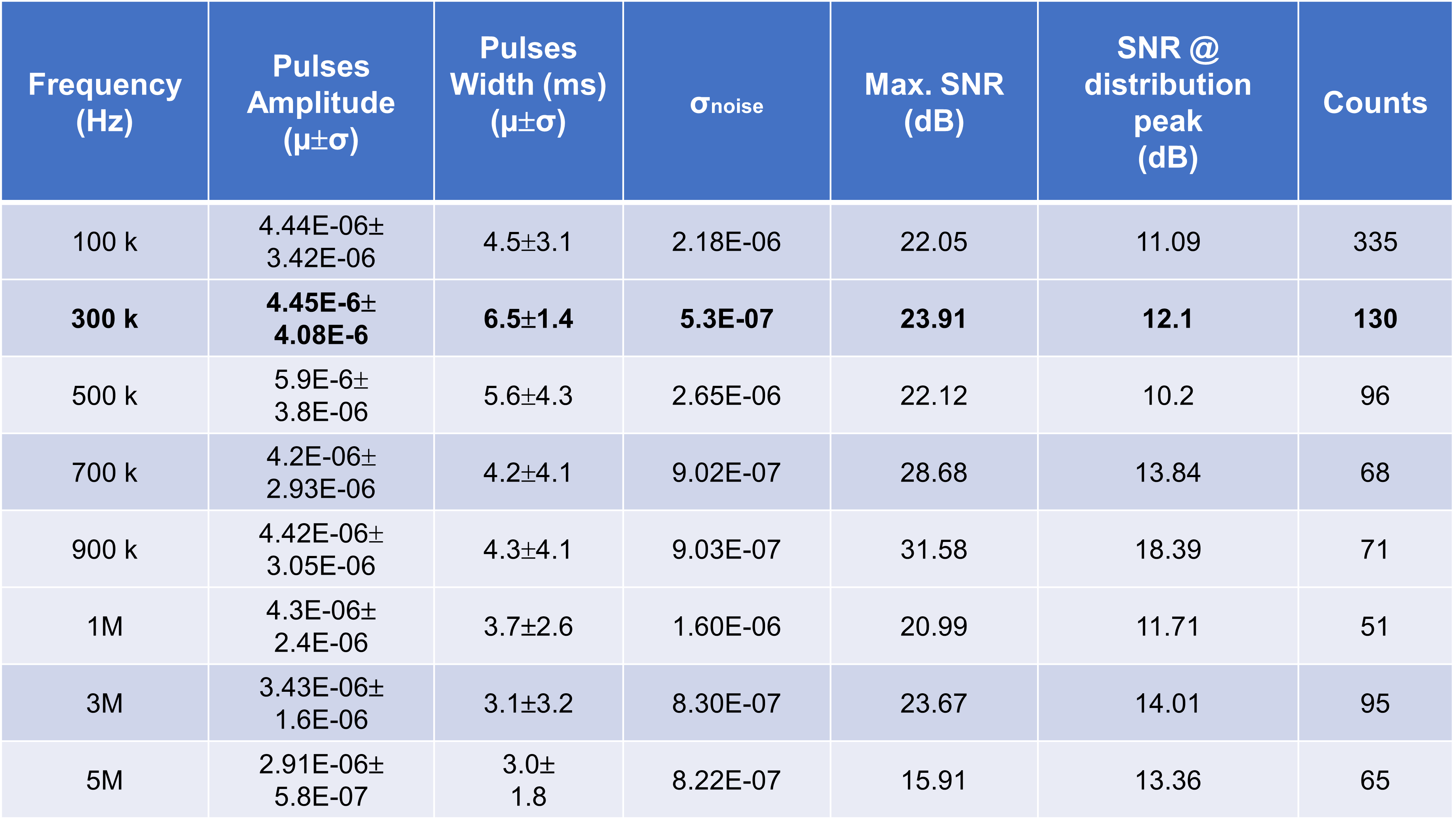


Table S7. Hemocytometer and biochip particle count per mL with particle diameter in µm, signal to noise (SNR) at maximum and mean point, experiment noise standard deviation, pulse amplitude, and average value with standard deviation are tabulated for Design M.

**
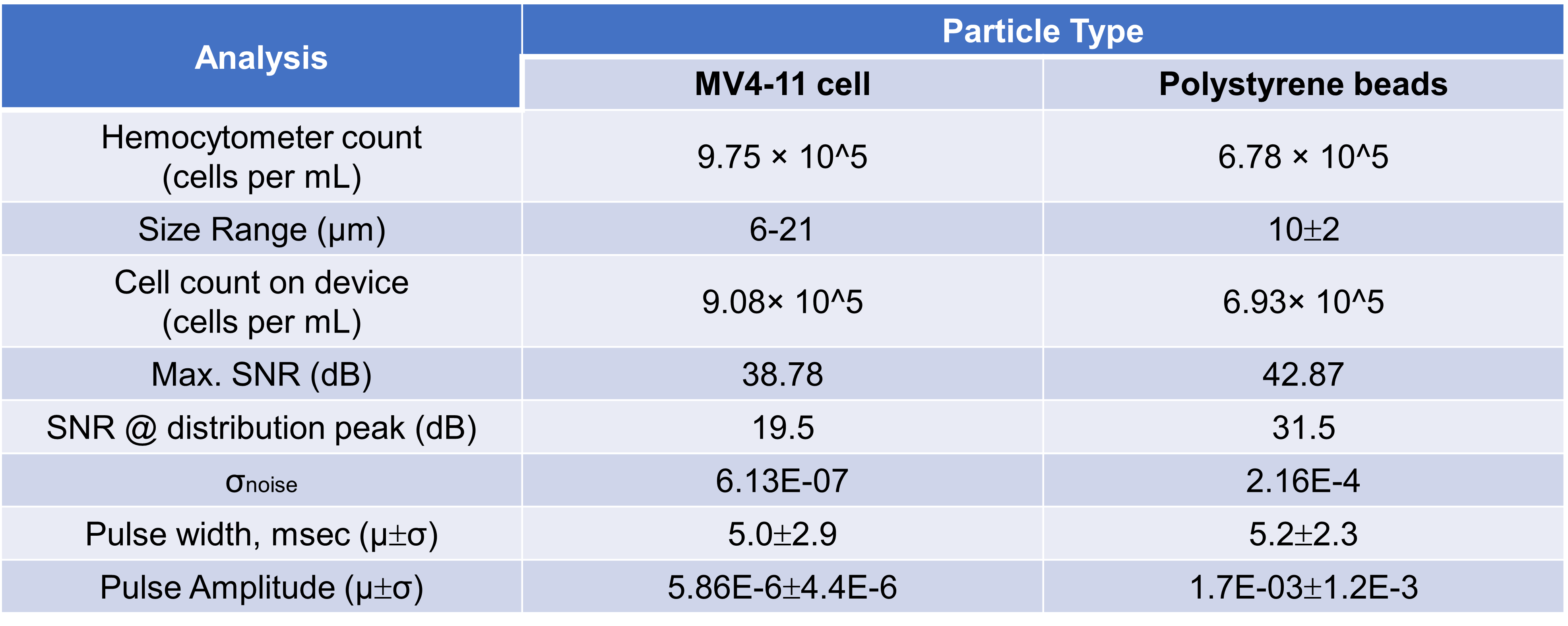
**

**References**

[1] G. H. Hsiue, P. L. Lu, and J. C. Chen, J. o. a. p. s."Multienzyme‐immobilized modified polypropylene membrane for an amperometric creatinine biosensor," vol. 92, no. 5, pp. 3126-3134, 2004.

[2] H.-T. Deng, Z.-K. Xu, J. Wu, P. Ye, Z.-M. Liu, and P. Seta, J. o. M. C. B. E."A comparative study on lipase immobilized polypropylene microfiltration membranes modified by sugar-containing polymer and polypeptide," vol. 28, no. 2-3, pp. 95-100, 2004.

[3] K. Kimura, T. Sugawara, M. Ebikawa, K. Kimura, J. Arisawa, and O. Igarashi, J. D. "Utilization of a newly established gene isolation technique with metal coating hollow fiber membrane for viral gene collection," vol. 149, no. 1-3, pp. 269-273, 2002.

[4] A. Okamura *et al.*, "Poly (N-isopropylacrylamide)-graft-polypropylene membranes containing adsorbed antibody for cell separation," vol. 26, no. 11, pp. 1287-1292, 2005.

[5] Q. Yang, M.-X. Hu, Z.-W. Dai, J. Tian, and Z.-K. Xu, J. L. "Fabrication of glycosylated surface on polymer membrane by UV-induced graft polymerization for lectin recognition," vol. 22, no. 22, pp. 9345-9349, 2006.

[6] S. K. McGraw, E. Alocilja, K. Senecal, and A. Senecal, "A resistance based biosensor that utilizes conductive microfibers for microbial pathogen detection," 2012.

[7] H. Wu *et al.*, "Carrying out pseudo dual nucleic acid detection from sample to visual result in a polypropylene bag with CRISPR/Cas12a," vol. 178, p. 113001, 2021.

[8] S. Iguchi *et al.*, "A flexible and wearable biosensor for tear glucose measurement," vol. 9, no. 4, pp. 603-609, 2007.

[9] M. M. Hasan *et al.*, "Functionalization of polypropylene nonwoven fabrics using cold plasma (O2) for developing graphene-based wearable sensors," vol. 300, p. 111637, 2019.
